# Prolonging valley polarization lifetime through gate-controlled exciton-to-trion conversion in monolayer molybdenum ditelluride

**DOI:** 10.1038/s41467-022-31672-y

**Published:** 2022-07-14

**Authors:** Qiyao Zhang, Hao Sun, Jiacheng Tang, Xingcan Dai, Zhen Wang, Cun-Zheng Ning

**Affiliations:** 1 Department of Electronic Engineering, Tsinghua University, 100084 Beijing, China; 2 Frontier Science Center for Quantum Information, 100084 Beijing, China; 3 Tsinghua International Center for Nano-Optoelectronics, Tsinghua University, 100084 Beijing, China

**Keywords:** Two-dimensional materials, Two-dimensional materials, Electronic and spintronic devices

## Abstract

Monolayer 2D semiconductors provide an attractive option for valleytronics due to valley-addressability. But the short valley-polarization lifetimes for excitons have hindered potential valleytronic applications. In this paper, we demonstrate a strategy for prolonging the valley-polarization lifetime by converting excitons to trions through efficient gate control and exploiting the much longer valley-polarization lifetimes for trions than for excitons. At charge neutrality, the valley lifetime of monolayer MoTe_2_ increases by a factor of 1000 to the order of nanoseconds from excitons to trions. The exciton-to-trion conversion changes the dominant depolarization mechanism from the fast electron-hole exchange for excitons to the slow spin-flip process for trions. Moreover, the degree of valley polarization increases to 38% for excitons and 33% for trions through electrical manipulation. Our results reveal the depolarization dynamics and the interplay of various depolarization channels for excitons and trions, providing an effective strategy for prolonging the valley polarization.

## Abstract (web summary)

Here, the authors devise a strategy for prolonging the valley polarization lifetime in monolayer MoTe_2_ by converting excitons to trions through gate control, and by taking advantage of the longer valley polarization lifetime of trions.

## Introduction

Valleytronics, in analogy to spintronics, aims to control and explore the valley degree of freedom (VDOF) analogously to the spin degree of freedom for information processing^1–7^. This field has been invigorated by the emergence of monolayer transition metal dichalcogenides (ML-TMDCs), which possess direct bandgaps at degenerate K/K’ valleys with the locked spin orientations. Thus, optical transitions in a specific valley can be selectively excited by circularly polarized light^5,8^. This permits the addressability and control of VDOF by optical means, providing an important potential for processing information stored in VDOF. However, this theoretical potential has encountered practical challenges. This is because optical transitions in TMDCs are dominated by excitons. But the valley polarization of excitons has a very short lifetime on the orders of a few picoseconds (ps)^9,10^, making exciton-based valleytronics impractical. The situation is even worse for Mo-based TMDCs such as MoTe_2_, due to the small energetic difference and the associated couplings between dark and bright excitons^11^. Trion (T), an excitonic complex consisting of an exciton (X) and an extra charge, can also stably exist in TMDCs^12,13^ with binding energies between 20 and 40 meV. Compared to excitons, the valley-polarization lifetime of trions has been proven to be much longer, ranging from tens to hundreds of ps^10,14,15^. A simple comparison of valley-polarization lifetimes of excitons and trions suggests a strategy of converting excitons to trions to prolong the valley polarization. Indeed, gate-controlled exciton-to-trion (X–T) conversions have been explored to realize optical gain associated with trion-electron population inversion at low levels of carrier densities^16^. Similar gate-controlled X–T conversion could enable long valley polarization so that VDOF of trions could be explored for information applications, such as electrically controlled logic gates in quantum computing^17,18^.

The electrical control of valley polarization has been studied in many TMDCs. The electron–hole (e–h) exchange interaction, the main depolarization mechanism of excitons, decreases with electric field^19^ and with electrical gating^20,21^ due to the Coulomb screening. Besides, the mutual conversion between excitons and trions plays an important role in the generation and manipulation of VDOF. For instance, the X–T conversion is an important decay channel for excitons^22,23^, affecting the lifetime of excitons. Interestingly, the intervalley scattering of excitons leads to fast depolarization of trions due to the X–T conversion in the opposite valley in an intrinsically doped ML-WSe_2_^14^. To manipulate valley polarization, chemical doping^24^ and electrical gating^25^ were considered to control the X–T conversion in continuous-wave photoluminescence (CW-PL) measurement. However, it is still unclear how the mutual conversion of excitons and trions, especially the interplay of their depolarization processes would affect the valley polarization dynamically. Furthermore, how gate control can be employed to effectively prolong the valley polarization maintenance time? Thus, ultrafast time-resolved measurement involving the X–T conversion process is necessary. Moreover, in-depth understandings of the time scales of various dynamical processes of excitons and trions, such as formation, conversion, and depolarization, provide fruitful experimental evidence and shed light on the mechanisms of valley generation and manipulation.

In this paper, we systematically investigate the valley polarization and valley dynamics in ML-MoTe_2_ using CW-PL and helicity-resolved pump–probe spectroscopy. Through the near-resonant excitation, we demonstrate the successful observation of PL polarization without a magnetic field in electrically gated ML-MoTe_2_. Moreover, we show that the PL polarization and depolarization times can be effectively controlled by the gate voltage through different depolarization mechanisms. The efficient X–T conversion changes the fast e–h exchange for excitons to the much slower spin-flip for trions. The generation, manipulation, and depolarization mechanisms of valley polarizations in ML-MoTe_2_ elucidated in this work enrich the understanding of the inner relationship between many-body interactions and valley dynamics.

Of special interest is our focus on MoTe_2_. To date, valley polarization has been mostly observed in the visible wavelength range^5,26,27^, while near-infrared valley polarization^28^ is the basic requirement for integrating valleytronic devices on the Silicon platform. ML-MoTe_2_ is an advantageous TMDC material, due to the silicon-transparent emission of excitonic transitions at room temperature, appealing to on-chip optoelectronic applications such as nanolasers^29^, light-emitting diodes, and detectors^30,31^. However, due to the strong e–h exchange effect^14,32^ and efficient couplings between dark and bright excitons^11^, the realization of valley polarization of excitons in ML-MoTe_2_ has proven to be very challenging^33^. For instance, usually a giant magnetic field is required^34–36^, which hinders the on-chip integration of valleytronic devices^28^. Our work paves the way for valleytronic applications integrated on the silicon platform.

## Results

### Gate-dependent PL valley polarizations

**Fig. 1:**
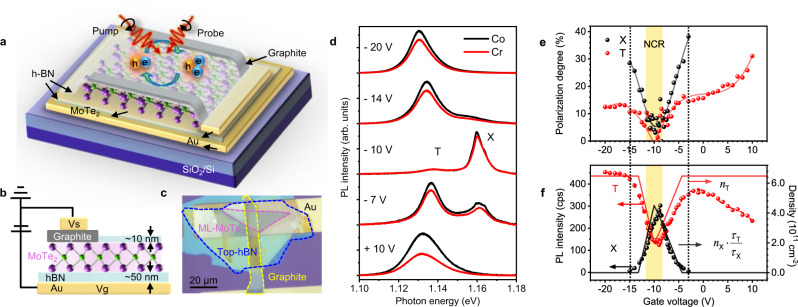
Sample structure and gate-tunable PL valley polarization. **a** Schematic diagram of the electrically gated ML-MoTe_2_ device. The ML-MoTe_2_ with top graphite electrode is encapsulated by thin layers of h-BN. And this sandwiched structure is placed on top of an Au/Ti electrode on a SiO_2_/Si substrate. **b** Cross-section diagram of the electrically gated structure. **c** Optical microscope image of a representative device (Device #1). **d** The helicity-resolved CW-PL emission of Device #1 at varying gate voltages with Δ*E* of 28 meV. The black and red lines denote the Co- and Cross (Cr) -circularly polarized emission with respect to the polarization direction of the pump laser. **e** The gate-dependent DVP extracted from the CW-PL measurement and **f** the gate-dependent PL emission intensity (dots) and the corresponding ratio of carrier densities to lifetimes (solid lines) of excitons (black) and trions (red). *n*_X_ (*n*_T_) and *τ*_X_ (*τ*_T_) represent the density and lifetime of excitons (trions). The dashed lines are fitted by polynomial functions to show the trends of PL intensity varying with gate voltages. The neutrally charged region (NCR) is shaded in yellow. The black dashed lines indicate the critical voltage range within which the exciton emission can be clearly resolved.

To investigate the valley polarization of ML-MoTe_2_, we utilized an electrically gated structure, as shown in Fig. 1a–c (see “Methods” for details). Based on the extensive studies of depolarization mechanisms in other ML-TMDCs^27,37,38^, we note that the near-resonant excitation is crucial to studying the valley polarization of excitons in ML-MoTe_2_ (Supplementary Note 1). In ML-MoTe_2_, the bright exciton has lower transition energy than the dark one with the conduction band splitting (*∆*_*c*_) of ~58 meV^33^, among the largest in TMDC materials. The excess energy (∆*E*), is defined as the detuning between pump energy and exciton resonance. In the CW-PL experiment, ∆*E* (~28 meV) is much smaller than *∆*_*c*_, meaning that only bright excitons and trions are generated by the pump. (see Supplementary Note 2 for hole– and electron–trion configurations in ML-MoTe_2_) And the small ∆*E* may generate negligible longitude acoustic phonons, indicating the phonon-assisted intervalley scattering can be neglected in our experiments. (Supplementary Note 1).

Figure 1d shows the gate-dependent helicity-resolved CW-PL at 4 K. The MoTe_2_ sample (Device #1) is intrinsically negatively charged and is electrically neutral at the gate voltage (Vg) of −10 V where the PL shows almost no trion emission. The PL emission intensities for the two polarizations are almost identical at −10 V, indicating a relatively low degree of valley polarization (DVP) even under near-resonant excitation. This suggests that the strong e–h exchange^39^ is the dominant depolarization mechanism in ML-MoTe_2_, as evidenced by the DVP results as a function of ∆*E*  shown in Supplementary Note 3. The DVP value is calculated as *P* = (Co-Cr)/(Co+Cr), where Co and Cr (Cross) is PL intensities in the same or opposite circular polarization with the pump laser. Figure 1e indicates that both excitons and trions show negligible DVP in the neutrally charged region (NCR). As the density of electrostatic background charges increases, the DVP increases significantly. This gate-dependent behavior will be explained in more detail in connection with time-resolved measurements shown below. The DVP of excitons increases faster and reaches saturation (with a maximum value of 38% at −3 V) earlier than that of trions. While the trion DVP continuously increases with increasing background charges and reaches a maximum value of 33%, which is comparable to the result of 36% measured under a magnetic field of 29 T^34^. And this type of enhancement of DVP by electrical gating in ML-MoTe_2_ was commonly observed in different samples in our experiments (Supplementary Note 4).

The gate-dependent PL emission intensity is plotted together with the ratio of carrier density to recombination lifetime in Fig. 1f. The densities of excitons and trions can be estimated through the steady-state solutions of the rate equations (see Supplementary Note 5) with the mass-action law. We pointed out that the mass-action law alone would be a poor approximation or even wrong if other processes are more important, as was the case in Ref. ^40^. But the rate equations, if correctly constructed, are more generally applicable to non-equilibrium cases where the X–T conversion term is one among many other processes. In the hole-doped region, the calculated carrier densities agree well with the gate-dependent PL emission intensities. The PL of trions is more sensitive to electron doping, possibly due to the intrinsic defect doping, leading to the fast saturation of PL emission with increasing electron density. In the NCR, the exciton emission is much stronger than that of trions. The strong depolarization of excitons leaves few excitons to convert to trions. The majority of trions are converted from the depolarized excitons in the opposite valley. Thus the DVP for both excitons and trions is low in the NCR. As the background carrier density increases, there are enough carriers to combine with excitons to form trions, and the trion density gradually surpasses exciton density. Fewer excitons are depolarized and the DVP of both excitons and trions increases. Far away from NCR, excitons are completely converted to trions. The PL of excitons is barely observable. This explains that exciton data in Fig. 1e extends to a much smaller range of gate voltages. The background carriers can also be polarized through the formation and intervalley scattering of trions. The DVP of background carriers is calculated up to 20% with the increase of carrier density. The calculation procedure and related discussion are presented in detail in Supplementary Note 6.

To gain more insight into the mechanisms of generation and manipulation of valley polarization, the following phenomenological valley-resolved rate equations were utilized:

1a $$\frac{{{{{{\rm{d}}}}}}{n}_{{{{{{\rm{X}}}}}}}^{{{{{{\rm{K}}}}}}/{{{{{\rm{K}}}}}}^{\prime} }}{{{{{{\rm{d}}}}}}t}={g}^{{{{{{\rm{K}}}}}}/{{{{{\rm{K}}}}}}^{\prime} }-{n}_{{{{{{\rm{X}}}}}}}^{{{{{{\rm{K}}}}}}/{{{{{\rm{K}}}}}}^{\prime} }{\Gamma }_{{{{{{\rm{X}}}}}}}^{{{{{{\rm{r}}}}}}}-{n}_{{{{{{\rm{X}}}}}}}^{{{{{{\rm{K}}}}}}/{{{{{\rm{K}}}}}}^{\prime} }{\Gamma }_{{{{{{\rm{XT}}}}}}}+\left({n}_{{{{{{\rm{X}}}}}}}^{{{{{{\rm{K}}}}}}^{\prime} /{{{{{\rm{K}}}}}}}-{n}_{{{{{{\rm{X}}}}}}}^{{{{{{\rm{K}}}}}}/{{{{{\rm{K}}}}}}^{\prime} }\right){\Gamma }_{{{{{{\rm{X}}}}}}}^{{{{{{\rm{sk}}}}}}}$$

1b $$\frac{{{{{{\rm{d}}}}}}{n}_{{{{{{\rm{T}}}}}}}^{{{{{{\rm{K}}}}}}/{{{{{\rm{K}}}}}}^{\prime} }}{{{{{{\rm{d}}}}}}t}=-{n}_{{{{{{\rm{T}}}}}}}^{{{{{{\rm{K}}}}}}/{{{{{\rm{K}}}}}}^{\prime} }{\Gamma }_{{{{{{\rm{T}}}}}}}^{{{{{{\rm{r}}}}}}}+{n}_{{{{{{\rm{X}}}}}}}^{{{{{{\rm{K}}}}}}/{{{{{\rm{K}}}}}}^{\prime} }{\Gamma }_{{{{{{\rm{XT}}}}}}}+\left({n}_{{{{{{\rm{T}}}}}}}^{{{{{{\rm{K}}}}}}^{\prime} /{{{{{\rm{K}}}}}}}-{n}_{{{{{{\rm{T}}}}}}}^{{{{{{\rm{K}}}}}}/{{{{{\rm{K}}}}}}^{\prime} }\right){\Gamma }_{{{{{{\rm{T}}}}}}}^{{{{{{\rm{sk}}}}}}}$$

**Fig. 2:**
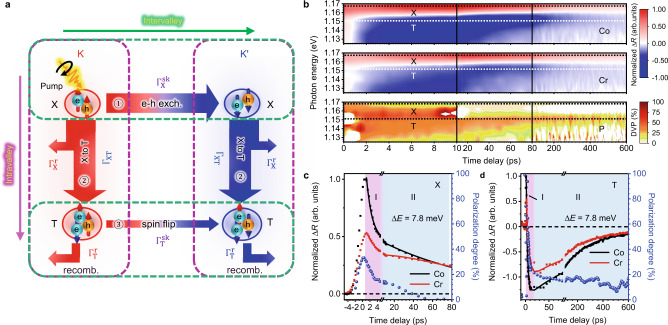
Decay processes and results of helicity-resolved pump–probe experiments. **a** Schematic diagram of different inter- and intravalley decay channels for excitons and trions. The colored arrows represent the flow of the carrier population. After initialized by σ^+^ pump, excitons in K valley face three outcomes: (1) scattered to K’ valley through e–h exchange interaction (intervalley process, ①); (2) converted to trions within K valley (②) and (3) recombine radiatively or non-radiatively. Both processes (2) and (3) happen within the same valley and do not contribute to depolarization. The trions in both valleys relax through two channels: (1) intervalley scattering to the other valley through spin-flip (intervalley process, ③); and (2) radiative or non-radiative recombinations within the same valley. **b** Transient differential reflectance spectra in colored contour map for Co- (top) and Cross- (Cr, middle) polarization with the corresponding polarization degree (P, bottom) of Device #2 in NCR (*V*_g_ = −13 V). The negative and positive signals in Co and Cross spectra correspond to the valley evolution dynamics of trions (T) and excitons (X), respectively. The co-polarized data (Co) reflects the decay processes within the excitation valley, while the cross-polarization signal (Cr) depicts the population redistribution dynamics between K and K’ valleys. **c**, **d** Near-resonant helicity-resolved differential reflectance signals of excitons (**c**) and trions (**d**) with excess energy of 7.8 meV for Device #3 at NCR, where the right axis (blue dots) shows the DVP obtained from the differential reflectance signals. The light purple and blue shadowed areas indicate the two stages of carrier evolution dynamics.

${n}_{{{{{{\rm{X}}}}}}}^{{{{{{\rm{K}}}}}}/{{{{{\rm{K}}}}}}^{\prime} }$ (${n}_{{{{{{\rm{T}}}}}}}^{{{{{{\rm{K}}}}}}/{{{{{\rm{K}}}}}}^{\prime} }$) represents the density of excitons (trions) in the K/K’ valley. ${g}^{{{{{{\rm{K}}}}}}/{{{{{\rm{K}}}}}}^{\prime} }$ is the generation rate of excitons. ${\Gamma }_{{{{{{\rm{X}}}}}}}^{{{{{{\rm{r}}}}}}}$ (${\Gamma }_{{{{{{\rm{T}}}}}}}^{{{{{{\rm{r}}}}}}}$) stands for the recombination rate of excitons (trions), and ${\Gamma }_{{{{{{\rm{XT}}}}}}}$ is the X–T conversion rate. Here we only consider trion formation through a bi-molecular process whereby an exciton captures an electron or a hole. The possibility of trimolecular formation of trions directly from photo-generated carriers is negligible, as discussed in Supplementary Note 7. The relaxation processes represented in Eqs. (1a) and (1b) are schematically depicted in Fig. 2a. These equations can be solved to obtain the DVP for excitons and trions, respectively, as

2a $${P}_{{{{{{\rm{X}}}}}}}={P}_{0}\frac{{\Gamma }_{{{{{{\rm{X}}}}}}}^{{{{{{\rm{ia}}}}}}}}{{\Gamma }_{{{{{{\rm{X}}}}}}}^{{{{{{\rm{ia}}}}}}}+{\Gamma }_{{{{{{\rm{X}}}}}}}^{{{{{{\rm{ir}}}}}}}}$$

2b $${P}_{{{{{{\rm{T}}}}}}}={P}_{{{{{{\rm{X}}}}}}}\frac{{\Gamma }_{{{{{{\rm{T}}}}}}}^{{{{{{\rm{ia}}}}}}}}{{\Gamma }_{{{{{{\rm{T}}}}}}}^{{{{{{\rm{ia}}}}}}}+{\Gamma }_{{{{{{\rm{T}}}}}}}^{{{{{{\rm{ir}}}}}}}}$$

where ${P}_{0}$ is the initial DVP for excitons, ${\Gamma }_{{{{{{\rm{X}}}}}}}^{{{{{{\rm{ia}}}}}}}$(${\Gamma }_{{{{{{\rm{T}}}}}}}^{{{{{{\rm{ia}}}}}}}$) and ${\Gamma }_{{{{{{\rm{X}}}}}}}^{{{{{{\rm{ir}}}}}}}$(${\Gamma }_{{{{{{\rm{T}}}}}}}^{{{{{{\rm{ir}}}}}}}$) denote the intravalley and intervalley decay rate of excitons (trions), respectively. The effective intravalley decay rate of excitons ${\Gamma }_{{{{{{\rm{X}}}}}}}^{{{{{{\rm{ia}}}}}}}$ is given by ${\Gamma }_{{{{{{\rm{X}}}}}}}^{{{{{{\rm{ia}}}}}}}={\Gamma }_{{{{{{\rm{X}}}}}}}^{{{{{{\rm{r}}}}}}}+{\Gamma }_{{{{{{\rm{XT}}}}}}}$. For trions, ${\Gamma }_{{{{{{\rm{T}}}}}}}^{{{{{{\rm{ia}}}}}}}={\Gamma }_{{{{{{\rm{T}}}}}}}^{{{{{{\rm{r}}}}}}}$, since the dissociation of trions into excitons can be neglected at low temperatures. The intervalley decay rate ${\Gamma }_{{{{{{\rm{X}}}}}}}^{{{{{{\rm{ir}}}}}}}$ (${\Gamma }_{{{{{{\rm{T}}}}}}}^{{{{{{\rm{ir}}}}}}}$) equals twice the intervalley scattering rate $2{\Gamma }_{{{{{{\rm{X}}}}}}}^{{{{{{\rm{sk}}}}}}}$($2{\Gamma }_{{{{{{\rm{T}}}}}}}^{{{{{{\rm{sk}}}}}}}$) (derivation details in Supplementary Note 8). For excitons, the initial polarization degree ${P}_{{{{{{\boldsymbol{0}}}}}}}$ is introduced to take into account the unavoidable scattering due to impurities or defects^32^. In reality, ${P}_{{{{{{\boldsymbol{0}}}}}}}$ is lower than 100%. The low DVP in neutrally gated ML-MoTe_2_ shown in Fig. 1d, e also stems from the low ratio of exciton intravalley decay rate to the total decay rate. The DVP for trions has a similar expression, except that its initial value is determined through the conversion of the polarized excitons. Therefore the DVP of trions is lower than that of excitons, which is consistent with the results in Fig. 1e.

The X–T conversion, as an important intravalley decay channel for excitons, plays an important role in DVP for both excitons and trions. Figure 2a shows the contributions of the X–T conversion to the increased DVP. As the increased carrier density accelerates the X–T conversion process, more excitons decay within the K valley rather than scattering to K’ valley, leading to the increase of ${P}_{{{{{{\rm{X}}}}}}}$ away from NCR. Moreover, as more excitons are converted to form trions, the probability of intervalley scattering via trions is increased. Since the intervalley decay rate of trions is much slower than that of excitons, the intervalley scattering of total populations is still greatly suppressed even when excitons are depleted. As a result, the trion DVP keeps increasing even without exciton emission. The electrical manipulation on X–T conversions can be seen in the relative intensity change and redistribution of excitons and trions in the gate-dependent CW-PL and absorption measurements of another device shown in Supplementary Note 9.

### Helicity-resolved pump–probe measurements

To investigate different mechanisms of valley depolarization, we performed helicity-resolved pump–probe spectroscopy on ML-MoTe_2_ with close-to-resonance excitation at 4 K. (see “Methods” for details) Importantly, the helicity-resolved time evolution of differential reflectance data ∆*R* allows us to determine important time constants of intra- and intervalley processes by fitting with the solutions of the valley-resolved rate equations (Supplementary Note 8). Figure 2b shows such differential reflectance mapping with pump energy of 1.181 eV (∆*E* = 15 meV) for Device #2, with the same structure as Device #1. The exciton and trion resonances can be identified in the broadband spectra, consistent with the absorption resonance identified by CW-PL and reflectance measurement. (see Supplementary Note 10 for the spectra fitting of exciton and trion energies in transient differential reflectance and CW measurement) The positive values near the exciton resonance (~1.166 eV) are due to the bleaching of exciton absorption. The negative peak at ~1.152 eV corresponds to trions as a result of bandgap renormalization with a pump-induced carrier density of ~3 × 10^12 ^cm^−2^ (see Supplementary Note 11 for the determination procedure of carrier densities). The clear difference between the Co and Cr signals indicates the unbalanced population evolution in K/K’ valleys. The corresponding DVP mapping labeled with P in Fig. 2b shows remarkably different dynamics of DVPs for excitons and trions. The exciton DVP reaches a maximum value of ~40% within the first few ps and quickly disappears within tens of ps, while the trion DVP almost maintains a constant value of ~20% for over 600 ps.

The different behaviors of DVP dynamics of excitons and trions can be further examined by varying both pump and probe energies. The detailed transient absorption results and related discussion of excitons and trions at different pump/probe energies are shown in Supplementary Note 12. The pump–probe experiments are performed at almost the exact resonances for excitons and trions respectively in neutrally gated Device #3, as shown in Fig. 2c, d. After initialized by the intense ${\sigma }^{+}$ circularly polarized pump, both the exciton and trion signals rise near zero time delay due to the bleaching of absorption as the formation of excitons and trions. Then the kinetic evolution of carriers can be divided into two stages, as shaded by the light purple (I) and blue (II) region. For the exciton polarization dynamics shown in Fig. 2c, a fast decay of the exciton population for both polarizations of stage I is attributed to the two processes occurring on similar time scales: the X–T conversion and the recombination process (${\Gamma }_{{{{{{\rm{X}}}}}}}^{{{{{{\rm{ia}}}}}}}={\Gamma }_{{{{{{\rm{X}}}}}}}^{{{{{{\rm{r}}}}}}}+{\Gamma }_{{{{{{\rm{XT}}}}}}}$). The intravalley lifetime $1/{\Gamma }_{{{{{{\rm{X}}}}}}}^{{{{{{\rm{ia}}}}}}}$ is fitted to be ~1.66 ± 0.1 ps. In stage II, the slow decay process is associated with the localized excitons possibly induced by deep-level defects. The extracted exciton DVP correspondingly reflects the depolarization process of free and localized excitons. The DVP reaches a maximum value of 33% and then decays in two similar stages: with the fast decay component assigned to the depolarization of free excitons, while the slow decay component stems from the localized excitons. Since the slow decay process typically occurs after the valley depolarization process of free excitons is completed, we will only focus on the time delay within stage I. Since the temporal resolution is much shorter than the pulse width of the pump laser (~500 fs), the short decay time which greatly influences the initial DVP of excitons, can not be clearly resolved. Within the decay process of free excitons, the DVP can be exponentially fitted to obtain the intervalley decay rate ${\Gamma }_{{{{{{\rm{X}}}}}}}^{{{{{{\rm{ir}}}}}}}$. The fitted $1/{\Gamma }_{{{{{{\rm{X}}}}}}}^{{{{{{\rm{ir}}}}}}}$ of ~0.85 ps is about one-half of the $1/{\Gamma }_{{{{{{\rm{X}}}}}}}^{{{{{{\rm{ia}}}}}}}$, leading to the relatively low PL DVP as indicated by Eq. (2a).

As for the trion polarization dynamics shown in Fig. 2d, the decreasing signals in stage I for both polarizations indicate the trion formation process from excitons^22^. This process is nearly on the same time scale as the free exciton decay. With the ${\sigma }^{+}$ excitation, the trions in K valley are formed mainly from free excitons excited in the K valley. While the trions in K’ valley are formed either by X–T conversion from intervalley scattered excitons in K’ valley, or by spin flipping of trions in K valley. As a result of rapid intervalley scattering of excitons and simultaneous X–T conversion, trions in K’ valley are more likely formed through the first way. The initial trion DVP attains the maximum value approaching 100%, due to the faster X–T conversion than the formation of trions in the opposite valley. Then the rapid decay of trion DVP is closely correlated with the X–T conversion in both valleys. After this fast decay process, the free excitons are almost completely depleted through either recombination or X–T conversion. In stage II, the trion populations start to decay, which can be well fitted with a biexponential function, corresponding to the recombination of mobile and immobile trions^41^. The trion DVP almost maintains at ~20% for over hundreds of ps, much longer than that of excitons. The spin flipping of the additional charge requires a large momentum and thus is a process of low probability, leading to the slow intervalley decay rate for trions.

### Manipulation of valley dynamics via X–T conversions

**Fig. 3:**
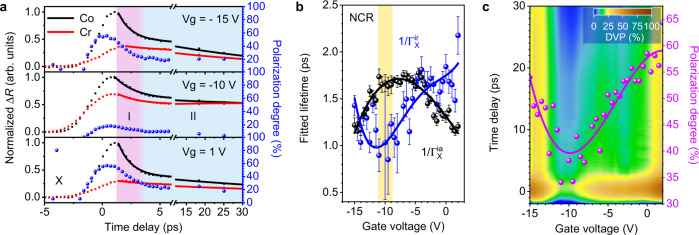
Electrical tuning of exciton valley dynamics. **a** Gate-dependent transient differential reflectance spectra for excitons with Δ*E* of 7.8 meV for Device #3 at three representative voltages: −15 V, −10 V, and 1 V, corresponding to the cases of positively charged, charge neutrality, and negatively charged, respectively. The black and red dots (solid lines) represent the measured data (exponential fit) of the Co and Cross (Cr) circularly polarized signal. The signals are normalized with respect to the intensity of the Co-polarized signal. The blue dots depict the dynamics of extracted DVP. **b** The gate-dependence of fitted intravalley decay time $1/{\Gamma }_{{{{{{\rm{X}}}}}}}^{{{{{{\rm{ia}}}}}}}$ (black dots), and intervalley decay times $1/{\Gamma }_{{{{{{\rm{X}}}}}}}^{{{{{{\rm{ir}}}}}}}$ (blue dots). The solid lines are polynomial fits. Error bars represent the uncertainty of lifetimes by exponential fitting. The decay variation of DVP at NCR is very small, leading to a large uncertainty of the exponential fitting of polarization decay. **c** The colored contour of DVP mapping in the plane of gate voltages and time delays, and the calculated DVP (magenta dots) for excitons using measured decay time in **b** by Eq. (2a) assuming *P*_0_ of 100%.

To study the valley depolarization process through X–T conversion, we investigated the gate-dependent polarization dynamics (see detailed electrically tunable results in Supplementary Note 13). Figure 3a displays the differential reflectance and evolution of DVP for excitons in Device #3 at three representative voltages. The maximum DVP is quite low (under 20%) in the case of charge neutrality (−10 V), while the DVP in the charged region can reach 60% initially and ~20% after 10 ps. The decay of DVP gets slower in the highly charged region, indicating the suppression of intervalley scattering of excitons. Figure 3b shows the fitted effective intravalley (intervalley) decay time $1/{\Gamma }_{{{{{{\rm{X}}}}}}}^{{{{{{\rm{ia}}}}}}}$ ($1/{\Gamma }_{{{{{{\rm{X}}}}}}}^{{{{{{\rm{ir}}}}}}}$) for excitons. In the NCR around −10 V, $1/{\Gamma }_{{{{{{\rm{X}}}}}}}^{{{{{{\rm{ia}}}}}}}$ shows a maximum value of 1.66 ± 0.1 ps, while $1/{\Gamma }_{{{{{{\rm{X}}}}}}}^{{{{{{\rm{ir}}}}}}}$ shows a minimum of 0.85 ± 0.37 ps. The generated excitons are prone to intervalley scattering and the DVP at −10 V has a maximum value of 18%. By tuning gate voltages away from NCR, $1/{\Gamma }_{{{{{{\rm{X}}}}}}}^{{{{{{\rm{ia}}}}}}}$ shows a slight decline, down to ~1.1 ps. Since the recombination rate of excitons changes negligibly with background charge density, the decrease of $1/{\Gamma }_{{{{{{\rm{X}}}}}}}^{{{{{{\rm{ia}}}}}}}$ is mainly due to the acceleration of the X–T conversion process, which competes with the intervalley scattering of excitons. Compared to the minor increase of intravalley lifetime, the intervalley decay time shows an increase as the voltage tunes away from the NCR. It more than doubles to ~2.17 ps at +2 V and is ~1.43 ps at −15 V. The increase of $1/{\Gamma }_{{{{{{\rm{X}}}}}}}^{{{{{{\rm{ir}}}}}}}$ is the result of a weakened e–h exchange interaction due to the increased screening from gate-generated charges.

Figure 3c shows the mapping of DVP decay with gate voltages. The maximum DVP increases significantly at time zero when the gate voltages are varied away from NCR, and the duration of DVP for excitons becomes longer. This directly shows the enhancement and prolonging of DVP for excitons with gate voltages away from NCR. The detailed measurement and fitting results of $1/{\Gamma }_{{{{{{\rm{X}}}}}}}^{{{{{{\rm{ia}}}}}}}$ at different gate voltages are shown in Supplementary Note 8. The exciton DVP can also be calculated through Eq. (2a) by using the measured decay rates in Fig. 3b. Assuming ${P}_{{{{{{\boldsymbol{0}}}}}}}$ to be 100% for simplicity, the value of ${\Gamma }_{{{{{{\rm{X}}}}}}}^{{{{{{\rm{ia}}}}}}}/({\Gamma }_{{{{{{\rm{X}}}}}}}^{{{{{{\rm{ia}}}}}}}+{\Gamma }_{{{{{{\rm{X}}}}}}}^{{{{{{\rm{ir}}}}}}})$ in Eq. (2a) increases with gate voltages varying away from NCR, which leads to the increased DVP for excitons. The DVP is ~35% at NCR, then increases to ~64% at 2 V and trends toward 100% with increased carrier density, as shown in Fig. 3c. However, the exciton emission decreases with charge density and is not observable at high charge densities. The calculated DVP in Device #3 shows a continuing increasing trend as in Device #1 shown in Fig. 1e, but the value is much higher, which is possibly attributed to the increased initial DVP caused by the increased pump fluence^21^. Although the pump-induced carrier density is different in our CW-PL (10^11 ^cm^−2^) and transient reflectance (10^12 ^cm^−2^) measurement, the intervalley relaxation rate shows little dependency on the pump fluence (see Supplementary Note 14).

**Fig. 4:**
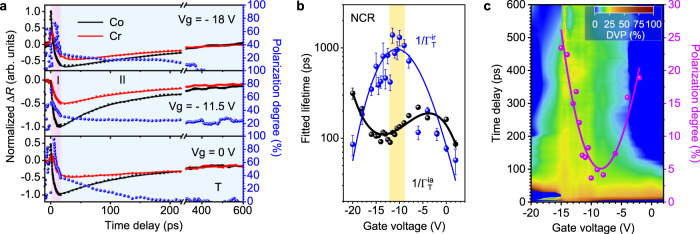
Electrical tuning of trion valley dynamics. **a** Gate-dependent transient differential reflectance spectra of trions with Δ*E* of 15 meV in Device #2. The extracted DVP for trions (blue dots) can be fitted by exponential functions after the depletion of free excitons. **b** The gate-dependence of fitted intravalley lifetime $1/{\Gamma }_{{{{{{\rm{T}}}}}}}^{{{{{{\rm{ia}}}}}}}$ (black dots), and intervalley decay time $1/{\Gamma }_{{{{{{\rm{T}}}}}}}^{{{{{{\rm{ir}}}}}}}$ (blue dots). The solid lines are polynomial fits, showing the data trends with gate voltages. Error bars represent the uncertainty of lifetimes by exponential fitting. **c** Colored contour of DVP for trions and the calculated DVP (magenta dots) using measured decay time in **b** from Eq. (2b).

Figure 4a shows the differential reflectance signal and the corresponding DVP for trions for Device #2. The two stages of trion population evolution, *i.e*. the formation and recombination process, show clear voltage dependency. In the NCR around – 11.5 V, the DVP for trions maintains at a nearly constant value of ~20% for over 600 ps. However, in highly charged situations, trions depolarize within ~200 ps. Figure 4b shows the extracted intravalley and intervalley decay time as a function of gate voltages. (representative fitting results in Supplementary Note 15) For Device #2, the trion population can be well fitted with a mono-exponential function due to the high quality of the sample. In the NCR case around −11.5 V, $1/{\Gamma }_{{{{{{\rm{T}}}}}}}^{{{{{{\rm{ia}}}}}}}$ shows a local minimum of ~90 ps. Then this value increases with the increased charge density, which is consistent with the theoretical results in Ref. ^42^, where it was attributed to the increasing difficulty of finding an unoccupied state in the conduction (valence) band with the increased charge density. However, this behavior is different from the cases of doped II-VI CdTe-based quantum wells, where the trion lifetime is carrier density insensitive^40^. At higher density above −5 V, the decrease of $1/{\Gamma }_{{{{{{\rm{T}}}}}}}^{{{{{{\rm{ia}}}}}}}$ is possibly due to the enhanced carrier interactions induced by the background population. The fitted $1/{\Gamma }_{{{{{{\rm{T}}}}}}}^{{{{{{\rm{ir}}}}}}}$ shows a value as high as ~1.39 ns in the NCR. This ultralong decay time is a result of the difficulty in finding a charge counterpart with an opposite spin in the NCR. With the increasing charge densities, it becomes easier to find such charge partners. Thus the spin flipping process of trions becomes faster, leading to the decrease of $1/{\Gamma }_{{{{{{\rm{T}}}}}}}^{{{{{{\rm{ir}}}}}}}$ to both sides. Other spin relaxation mechanisms, including Elliot–Yafet (EY), D’yakonov–Perel’ (DP), and Bir–Aronov–Pikus (BAP) mechanisms, might also contribute to the gate-dependent behaviors of spin relaxation^5,43^ (see discussions of different spin relaxation mechanisms in Supplementary Note 16).

It is interesting to view the trion DVP in the CW experiments presented in Fig. 1e from the perspectives of the various decay processes after these decay rates were determined. In the NCR in Fig. 4b, $1/{\Gamma }_{{{{{{\rm{T}}}}}}}^{{{{{{\rm{ir}}}}}}}$ is approximately one order of magnitude longer than $1/{\Gamma }_{{{{{{\rm{T}}}}}}}^{{{{{{\rm{ia}}}}}}}$, leading to a large value of ${\Gamma }_{{{{{{\rm{T}}}}}}}^{{{{{{\rm{ia}}}}}}}/({\Gamma }_{{{{{{\rm{T}}}}}}}^{{{{{{\rm{ia}}}}}}}+{\Gamma }_{{{{{{\rm{T}}}}}}}^{{{{{{\rm{ir}}}}}}})$ to be ~0.9 and ${P}_{{{{{{\rm{T}}}}}}}=0.9{P}_{{{{{{\rm{X}}}}}}}$ from Eq. (2b). Moreover, the exciton DVP ${P}_{{{{{{\rm{X}}}}}}}$ in CW-PL is low in NCR in Fig. 1e, leading to the negligible DVP for trions despite the ultralong trion intervalley decay time shown in Fig. 4b. In the case of high charge density, $1/{\Gamma }_{{{{{{\rm{T}}}}}}}^{{{{{{\rm{ir}}}}}}}$ decreases to the same scale as $1/{\Gamma }_{{{{{{\rm{T}}}}}}}^{{{{{{\rm{ia}}}}}}}$, which is not favorable for maintaining trion polarization. However, as the X–T conversion is enhanced, more excitons convert to trions due to the suppressed e–h exchange, resulting in the increase of the initial DVP for trions.

Taking the limited exciton DVP values within the black dashed region in Fig. 1e as the initial trion DVP and using the fitted intra- and intervalley decay rate in Fig. 4b, we can calculate the DVP for trions using Eq. (2b), as shown in Fig. 4c. The calculated DVP for trions is ~5% at −11 V and reaches ~24% at −15 V and ~19% at −2 V. Although $1/{\Gamma }_{{{{{{\rm{T}}}}}}}^{{{{{{\rm{ir}}}}}}}$ becomes shorter with charge density and is comparable to $1/{\Gamma }_{{{{{{\rm{T}}}}}}}^{{{{{{\rm{ia}}}}}}}$ at higher charge density, the trion polarization ${P}_{{{{{{\rm{T}}}}}}}$ continuously increases with increasing charge density due to the enhanced X–T conversion. The electrical control on X–T conversions not only affects the exciton decay dynamics, but also plays an important role in the intervalley scattering process of both excitons and trions, leading to an effective generation of valley polarization. Moreover, the DVP maintenance time is dramatically improved from a few ps of excitons to hundreds of ps of trions.

**Fig. 5:**
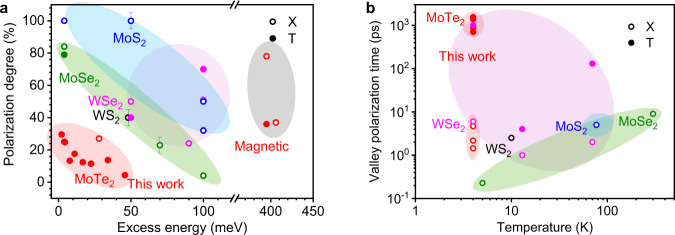
Comparison of valley polarization and intervalley scattering time with other ML-TMDCs. **a** Comparison of CW-PL DVP as a function of the excess energy at low temperatures (4–125 K)^5,6,27,37,44,45^. **b** Comparison of intervalley scattering time at different temperatures^9,10,15,46–49^. The colored circles represent different TMDCs.

Figure 5a, b shows the CW-PL polarization and intervalley scattering time of ML-MoTe_2_ in comparison with the corresponding quantities of other ML-TMDCs. The detailed test conditions, DVP values^5,6,27,37,44,45^, and intervalley scattering times^9,10,15,46–49^ are summarized in Supplementary Note 17. The DVP values of other ML-TMDCs exceed 40% and generally decrease rapidly with increasing ∆*E*, while MoS_2_ reaches close to unity polarization under near-resonant excitation. Through gating, the DVP value of ML-MoTe_2_ ranges from 0 to 38% for excitons and 33% for trions. At low temperatures from 4 to 125 K, the exciton intervalley scattering times in different ML-TMDCs range from 1 to 9 ps. And the trion scattering times are typically one order of magnitude longer than that for excitons^10,15^. Our results show that $1/{\Gamma }_{{{{{{\rm{X}}}}}}}^{{{{{{\rm{ir}}}}}}}$ is in the range of 1–4 ps and $1/{\Gamma }_{{{{{{\rm{T}}}}}}}^{{{{{{\rm{ir}}}}}}}$ is within 0.9–1.5 ns, which is much longer than for the other ML-TMDCs. The long valley lifetime of trions is a key parameter for information storage, and our results will help make valleytronic devices more practically likely.

## Discussion

Spin-valley physics and related device applications have been at the center of TMDCs research. In this work, through near-resonant excitation, we showed that the DVP of ML-MoTe_2_ at 4 K can be controllably increased by gating from near zero to 38% and 33% for excitons and trions, respectively. The maximum DVP of trions in ML-MoTe_2_ in the absence of a magnetic field is comparable to the value obtained under a magnetic field of 29 T^34^. In addition, gate-dependent valley dynamics of both excitons and trions were systematically investigated to elucidate how many-body interactions affect valley depolarization. The rapid X–T conversion preserve and prolong the valley polarization by converting the fast depolarization mechanism of excitons through e–h exchange interaction to the much slower spin-flip of trions, especially in the situation of charge neutrality. An ultralong valley polarization maintenance time exceeding 600 ps was observed for trions. This could be beneficial for the detection and manipulation of valley-related effects such as valley Hall effect^50^, and other valleytronic applications. The valley lifetime can be further extended by using TMDC heterostructures, for example, by exploiting the large separation of the wave functions of electrons and holes in interlayer excitons. By integrating with chiral metasurfaces or photonic structures, the valley index can be efficiently controlled, detected, and manipulated over long-time scales. The understanding of many-body effects on spin-valley polarizations may also stimulate further research on related issues in condensed matter physics of 2D materials, such as twistronics and related applications. Our results will enrich the understanding of valley dynamics in ML-TMDCs and may contribute to future on-chip valleytronic applications in the near-infrared wavelengths using MoTe_2_, given the predominant role of Silicon in current electronic and spintronic applications.

## Methods

### Fabrication of electrically gated ML-MoTe_2_ devices

The MoTe_2_ MLs, hexagonal boron nitride (h-BN), and graphite films were mechanically exfoliated from commercial bulk crystals (2D semiconductors or HQ graphene Inc.). The exfoliated flakes were transferred onto polydimethylsiloxane (PDMS) stamps by the dry transfer method. The layer thickness is identified by atomic force microscopy and contrast of optical microscope images. The ML-MoTe_2_ is encapsulated by h-BN to enhance the material quality. The electrodes of the gated structures were predefined by photolithography on a Si substrate with 300 nm SiO_2_ and then deposited with 50/30 nm Au/Ti by electron beam evaporation. After fabrication of the electrodes, a thin h-BN film of ~50 nm thickness and ML-MoTe_2_ were sequentially transferred onto the back gate electrode by the employment of micromanipulators for precise alignment. A graphite stripe of ~10 nm thickness was then transferred as a top contact bridging between MoTe_2_ and the other Au/Ti electrode. A second h-BN film of ~10 nm thickness was transferred on top of the device for protection from contamination. All the transfer processes were carried out with the aid of PDMS as carrier stamp at moderate heating temperature to avoid possible sample degradation. Finally, the device was annealed at ~200 °C for 3 h to reduce the air gaps between layers.

### Steady-state optical spectroscopy

Steady-state optical properties of ML-MoTe_2_ are characterized in a home-build micro-PL system at a cryogenic temperature of 4 K. A 632.8 nm continuous-wave HeNe laser or tunable diode laser (980–1060 nm, Toptica DL 100) was used as the pumping source for PL measurement. A combination of several sharp-edge long-pass filters was used for near-resonant excitation. For CW-reflectance measurements, a stabilized tungsten halogen lamp (Thorlabs SLS201) was used as the broadband source. The laser or white light excites the sample through a 100x NIR-optimized objective with NA = 0.7. The reflected signal was collected by the same objective and delivered to a spectrometer (Princeton Instruments Acton 2560i) equipped with LN-cooled InGaAs CCD for detection. The spot size of the pump laser was estimated to be ~3 µm in diameter using the knife-edge method. Electrical gating was conducted by using a commercial source meter (Keysight 2902A) or a semiconductor parameter analyzer (Keysight B1500A).

### Helicity-resolved pump–probe spectroscopy

Helicity-resolved ultrafast pump–probe spectroscopy was carried out using a 1040 nm femtosecond laser (pulse width of ~500 fs, repetition rate of 400 kHz, Spirit from Spectra Physics). The laser was divided into pump and probe beams. The near-resonant pump excitation was achieved through optical parameter amplifier (OPA) system with carrier density estimated to be at ~10^12 ^cm^−2^. The probe pulse is spectrally broadened by the white-light generation setup, including a focusing lens and a sapphire crystal. Both pump and probe beams are modulated by a mechanical chopper. A linear polarizer and λ/4 wave plate are used for circular polarization control of pump and probe pulses with ~95% circularity. Both beams are combined with a non-polarized beamsplitter and sent to a 50x objective with NA = 0.4. With grating-based pulse shapers, pump and probe beams can be spectrally filtered with FWHM of ~3 meV. The energy difference below 10 meV between pump and probe can be well resolved by the spectrometer. The differential reflectance signal $\varDelta R$ is defined as Δ*R* = *R*_w_−*R*_wo_ where *R*_w(wo)_ is the reflected probe intensity with (without) pump and detected by an InGaAs detector using the lock-in technique. The transient differential reflectance Δ*R*, the increase of the reflection intensity of probe pulse induced by pumping, is proportional to the negative absorption, $\varDelta R\propto -\alpha (0)$.

## Supplementary information

Supplementary Information

## Data Availability

All data needed to evaluate the conclusions in the paper are present in the main text and the supplementary materials. Additional data related to this paper may be requested from the authors upon reasonable request.

## References

[1] Rycerz A, Tworzydło J, Beenakker CWJ (2007). Valley filter and valley valve in graphene. Nat. Phys..

[2] Akhmerov AR, Beenakker CWJ (2007). Detection of valley polarization in graphene by a superconducting contact. Phys. Rev. Lett..

[3] Yao W, Xiao D, Niu Q (2008). Valley-dependent optoelectronics from inversion symmetry breaking. Phys. Rev. B.

[4] Xiao D (2012). Coupled spin and valley physics in monolayers of MoS_2_ and other group-VI dichalcogenides. Phys. Rev. Lett..

[5] Mak KF, He K, Shan J, Heinz TF (2012). Control of valley polarization in monolayer MoS_2_ by optical helicity. Nat. Nanotechnol..

[6] Zeng H, Dai J, Yao W, Xiao D, Cui X (2012). Valley polarization in MoS_2_ monolayers by optical pumping. Nat. Nanotechnol..

[7] Cao T (2012). Valley-selective circular dichroism of monolayer molybdenum disulphide. Nat. Commun..

[8] Ye Z, Sun D, Heinz TF (2017). Optical manipulation of valley pseudospin. Nat. Phys..

[9] Ye Y (2016). Electrical generation and control of the valley carriers in a monolayer transition metal dichalcogenide. Nat. Nanotechnol..

[10] Yan T, Yang S, Li D, Cui X (2017). Long valley relaxation time of free carriers in monolayer WSe_2_. Phys. Rev. B.

[11] Yang M (2020). Exciton valley depolarization in monolayer transition-metal dichalcogenides. Phys. Rev. B.

[12] Chernikov A (2014). Exciton binding energy and nonhydrogenic Rydberg series in monolayer WS_2_. Phys. Rev. Lett..

[13] Wang G (2018). Colloquium: Excitons in atomically thin transition metal dichalcogenides. Rev. Mod. Phys..

[14] Wang G (2014). Valley dynamics probed through charged and neutral exciton emission in monolayer WSe_2_. Phys. Rev. B.

[15] Singh A (2016). Long-Lived Valley Polarization of Intravalley Trions in Monolayer WSe_2_. Phys. Rev. Lett..

[16] Wang Z (2020). Excitonic complexes and optical gain in two-dimensional molybdenum ditelluride well below the Mott transition. Light Sci. Appl..

[17] Ang YS, Yang SA, Zhang C, Ma Z, Ang LK (2017). Valleytronics in merging Dirac cones: all-electric-controlled valley filter, valve, and universal reversible logic gate. Phys. Rev. B.

[18] Pawłowski J, Zebrowski D, Bednarek S (2018). Valley qubit in a gated MoS_2_ monolayer quantum dot. Phys. Rev. B.

[19] Chakraborty C, Mukherjee A, Qiu L, Vamivakas AN (2019). Electrically tunable valley polarization and valley coherence in monolayer WSe_2_ embedded in a van der Waals heterostructure. Opt. Mater. Express.

[20] Shinokita K (2019). Continuous control and enhancement of excitonic valley polarization in monolayer WSe_2_ by electrostatic doping. Adv. Funct. Mater..

[21] Feng S (2019). Engineering valley polarization of monolayer WS_2_: a physical doping approach. Small.

[22] Singh A (2016). Trion formation dynamics in monolayer transition metal dichalcogenides. Phys. Rev. B.

[23] Gao F (2016). Valley trion dynamics in monolayer MoSe_2_. Phys. Rev. B.

[24] Carmiggelt JJ, Borst M, van der Sar T (2020). Exciton-to-trion conversion as a control mechanism for valley polarization in room-temperature monolayer WS_2_. Sci. Rep..

[25] Hanbicki AT (2016). High room temperature optical polarization due to spin-valley coupling in monolayer WS_2_. AIP Adv..

[26] Zhu B, Zeng H, Dai J, Gong Z, Cui X (2014). Anomalously robust valley polarization and valley coherence in bilayer WS_2_. Proc. Natl Acad. Sci. USA.

[27] Kioseoglou G, Hanbicki AT, Currie M, Friedman AL, Jonker BT (2016). Optical polarization and intervalley scattering in single layers of MoS_2_ and MoSe_2_. Sci. Rep..

[28] Huang Z (2020). Robust room temperature valley Hall effect of interlayer excitons. Nano Lett..

[29] Li Y (2017). Room-temperature continuous-wave lasing from monolayer molybdenum ditelluride integrated with a silicon nanobeam cavity. Nat. Nanotechnol..

[30] Bie Y-Q (2017). A MoTe_2_-based light-emitting diode and photodetector for silicon photonic integrated circuits. Nat. Nanotechnol..

[31] Zhu Y (2018). High-efficiency monolayer molybdenum ditelluride light-emitting diode and photodetector. ACS Appl. Mater. Interfaces.

[32] Wang Q (2013). Valley carrier dynamics in monolayer molybdenum disulfide from helicity-resolved ultrafast pump-probe spectroscopy. ACS Nano.

[33] Robert C (2016). Excitonic properties of semiconducting monolayer and bilayer MoTe_2_. Phys. Rev. B.

[34] Arora A (2016). Valley Zeeman splitting and valley polarization of neutral and charged excitons in monolayer MoTe_2_ at high magnetic fields. Nano Lett..

[35] Jiang C (2017). Zeeman splitting via spin-valley-layer coupling in bilayer MoTe_2_. Nat. Commun..

[36] Smoleński T (2020). Valley pseudospin relaxation of charged excitons in monolayer MoTe_2_. J. Phys. Condens. Matter.

[37] Tornatzky H, Kaulitz AM, Maultzsch J (2018). Resonance profiles of valley polarization in single-layer MoS_2_ and MoSe_2_. Phys. Rev. Lett..

[38] Baranowski M (2017). Dark excitons and the elusive valley polarization in transition metal dichalcogenides. 2D Mater..

[39] Maialle MZ, De Andrada E Silva EA, Sham LJ (1993). Exciton spin dynamics in quantum wells. Phys. Rev. B.

[40] Kossacki P (2003). Optical studies of charged excitons in II-VI semiconductor quantum wells. J. Phys. Condens. Matter.

[41] Lampert MA (1958). Mobile and immobile effective-mass-particle complexes in nonmetallic solids. Phys. Rev. Lett..

[42] Wang H (2016). Radiative lifetimes of excitons and trions in monolayers of the metal dichalcogenide MoS_2_. Phys. Rev. B.

[43] Wang L, Wu MW (2014). Electron spin relaxation due to D’yakonov-Perel’ and Elliot-Yafet mechanisms in monolayer MoS_2_: Role of intravalley and intervalley processes. Phys. Rev. B.

[44] Lagarde D (2014). Carrier and polarization dynamics in monolayer MoS_2_. Phys. Rev. Lett..

[45] Wang G (2015). Polarization and time-resolved photoluminescence spectroscopy of excitons in MoSe_2_ monolayers. Appl. Phys. Lett..

[46] Zhu CR (2014). Exciton valley dynamics probed by Kerr rotation in WSe_2_ monolayers. Phys. Rev. B.

[47] Dal Conte S (2015). Ultrafast valley relaxation dynamics in monolayer MoS_2_ probed by nonequilibrium optical techniques. Phys. Rev. B.

[48] Kumar N, He J, He D, Wang Y, Zhao H (2014). Valley and spin dynamics in MoSe_2_ two-dimensional crystals. Nanoscale.

[49] Rodek A (2021). Local field effects in ultrafast light-matter interaction measured by pump-probe spectroscopy of monolayer MoSe_2_. Nanophotonics.

[50] Mak KF, McGill KL, Park J, McEuen PL (2014). The valley Hall effect in MoS_2_ transistors. Science.

