# Supplementary material for: Prolonging valley polarization lifetime through gate-controlled exciton-to-trion conversion in monolayer molybdenum ditelluride
Source: Nat Commun. 2022 Jul 14;13:4101. doi: 10.1038/s41467-022-31672-y (PMC9283389; doi:10.1038/s41467-022-31672-y)
Supplement: Supplementary file 1 — Supplementary Information [file 41467_2022_31672_MOESM1_ESM.pdf]

Supplementary Materials for

Prolonging Valley Polarization through Gate-Controlled Exciton-to-Trion Conversion in Monolayer Molybdenum Ditelluride

Qiyao Zhang,[1,2,3]† Hao Sun,[1,2,3]† Jiacheng Tang,[1,2,3] Xingcan Dai,[1] Zhen Wang,[1,2,3] Cun-Zheng Ning[1,2,3]*

† These authors contributed equally to this work

*Corresponding author. Email: cning@tsinghua.edu.cn

This PDF file includes:

Supplementary Notes 1 to 17

Supplementary Figures 1 to 15

Supplementary Tables 1 to 4

Supplementary References 1 to 35

List of Contents

Supplementary Note 1. Depolarization channels of excitons in ML-$MoTe_2$

Supplementary Note 2. Band structure and trion configurations in ML-$MoTe_2$

Supplementary Note 3. Influences of excess energy to polarization degree

Supplementary Note 4. CW-PL valley polarization of another device (Device #3)

Supplementary Note 5. Determination of gate-controlled exciton and trion density

Supplementary Note 6. Determination of DVP for background carriers

Supplementary Note 7. Possibility of trimolecular formation of trions in ML-$MoTe_2$

Supplementary Note 8. Phenomenological modeling of valley-resolved rate equations

Supplementary Note 9. Steady-state optical characterization

Supplementary Note 10. Spectra fitting of time-resolved differential reflectance

Supplementary Note 11. Pump induced carrier density in time-resolved pump-probe measurements

Supplementary Note 12. Pump (probe) energy dependent transient absorption spectroscopy

Supplementary Note 13. Detailed electrical tuning of valley polarization dynamics

Supplementary Note 14. Influence of photo-induced carrier density on the intervalley scattering

Supplementary Note 15. Fitting reliability of the valley dynamics

Supplementary Note 16. Calculation of spin relaxation time by DP and BAP mechanism

Supplementary Note 17. Comparison with other reports

Supplementary Note 1. Depolarization channels of excitons in ML-$MoTe_2$

The typical depolarization mechanisms, which have been studied previously in ML-TMDCs, includes: (i) phonon-assisted intervalley scattering; (ii) long-range electron-hole (e-h) exchange interactions, known as Maialle-Silva-Sham (MSS) mechanism [1]. The excess energy, defined as the detuning between pump energy and exciton resonance ($\Delta E = \hbar\omega - E_X$), plays an important role in these two depolarization channels. Here, $\Delta E = \hbar\omega - E_X$, where $\hbar\omega$ is the pump laser photon energy and E_X is the exciton emission energy. The phonon-induced intervalley scattering mechanism has been widely discussed for MoS_2 and $MoSe_2$ [2,3]. For this mechanism to be effective, a threshold limit for the detuning parameter, ΔE, equal to twice the longitude acoustic (LA) phonon energy $2E_{LA}$ is required to scatter both the electron and hole from K to K' valley at the same time. ML-$MoTe_2$ has a much smaller LA phonon energy of E_{LA} ~ 12 meV than other TMDCs [3,4]. This requires the pump photon energy to be very close to exciton energy. This might be one of the reasons that valley polarization was not observed in ML-$MoTe_2$ previously [4], where a ΔE of 60 meV was used. Although ΔE (28 meV) in CW-PL experiment is slightly larger than twice the longitude acoustic (LA) phonon energy ($2E_{LA}$), the small energy difference ($\Delta E - 2E_{LA}$) indicates the population of LA phonon (proportional to the phonon-assisted scattering rate), given by $\langle n \rangle = 1/(e^{2E_{LA}/(\Delta E\text{-}2E_{LA})} - 1)$ [2], is negligible. Thus the phonon-assisted intervalley scattering can be neglected in our experiments. Another important depolarization channel is the long-rage e-h exchange interaction of excitons. This e-h exchange interaction also highly relies on ΔE. Therefore, it is crucial to study the valley polarization of excitons in $MoTe_2$ system using near-resonant excitation

Supplementary Note 2. Band structure and trion configurations in ML-$MoTe_2$

In ML-$MoTe_2$, both the conduction and valence band has a large spin-splitting energy of 58 meV and 275 meV [4], respectively. Different from W-based TMDC, the bright exciton occupies the lowest energy transition due to negative conduction band splitting in ML-$MoTe_2$, as shown in Supplementary Figure 1 (a). Combining with an extra free carrier, the excitons (X) can convert to a bound state of trions (T). The formed trions inherit the same wave vector as the extra electrons

or holes. Thus the emission and absorption processes from the electronic state to the trion state are free of momentum change.

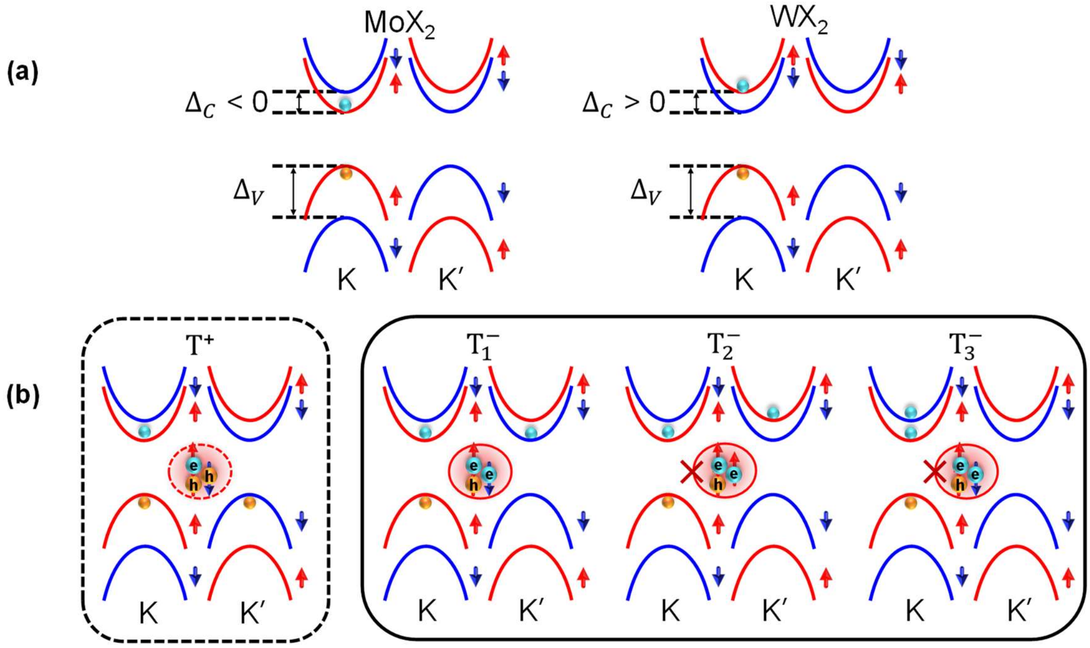

Supplementary Figure 1

Schematic illustration of trion configuration in ML-MoTe$_2$. (a) Schematic of the electronic band structure of Mo-based and W-based TMDC. Δ_C and Δ_V are the splitting of conduction and valance band, respectively. The arrows indicate the spin directions. (b) Schematic of hole-trion (dashed frame) and electron-trion (solid frame) configurations. Three configurations of electron trion including intervalley singlet trion (T_1^-), intervalley triplet trion (T_2^-) and intravalley singlet trion (T_3^-) are shown. Energies are not to scale for clarity.

As illustrated in Supplementary Figure 1 (b), in ML-MoTe$_2$, the hole-trion has the exclusive state due to the large valence band splitting, while for electron-trions, there might exist three possible configurations. Given the excitons are formed in K valley, the trion state with the lowest energy is the intervalley singlet trion labelled as T_1^-, with the extra electron located in the lower conduction band in K' valley. The free electrons may also occupy the higher conduction band as the carrier density increases, forming the intervalley triplet (T_2^-) and intravalley singlet trion (T_3^-). However, the trion emission in our measurements has a single peak without different trion species, as shown

in the mapping of PL emission with gate voltages in the following SM Section S8. And the determined background charge density is $10^{11} \sim 10^{12}$ cm^{-2}, as shown in more details in SM Section S4. The Fermi energy, $E_F = \pi\hbar^2 n_e / m_e$, where m_e is the effective electron mass for $MoTe_2$ [5], is well below the splitting of conduction band of 58 meV [4], indicating the trions with higher energy are not likely to exist in our system.

Supplementary Note 3. Influences of excess energy to polarization degree

In order to determine the main depolarization mechanism in ML-$MoTe_2$, the relation between intervalley scattering and excess energy is investigated by helicity-resolved pump-probe method. To obtain the degree of valley polarization (DVP) with resonant excitation, due to the filter bandwidth limitation, initial DVP of trions, rather than excitons is measured at different excess energies. The initial DVP is defined as the DVP calculated by the peak value in K and K' valley, which represents the maximum population in each valley. The excess energy is tuned from 0 to 23 meV, below twice of the LA phonon energy ($\Delta E < 2E_{LA} = 24$ meV) to exclude the influence of phonon-assisted intervalley scattering.

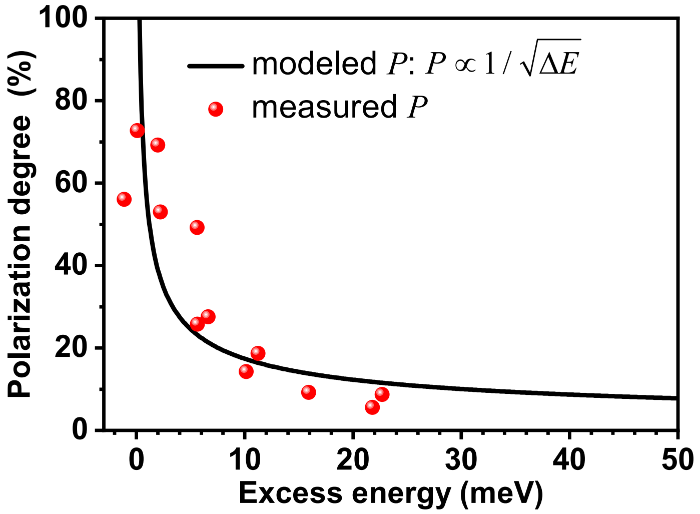

Supplementary Figure 2

Relationship between excess energy and initial DVP for trions in ML-$MoTe_2$. Initial trion DVP of measured data (red dots) fits well to excess energy model $P \propto 1/\sqrt{\Delta E}$ (black line).

To quantitatively analyze the inter-relations between excess energy and valley polarization, we used the excess energy model $P \propto 1/\sqrt{\Delta E}$ to fit experiment data [6]. The modeled curve shows good agreement with measured P_T below the 2LA phonon energy range, as shown in Supplementary Figure 2. The DVP decreases with increased excess energies, indicating that the accelerated intervalley scattering is mainly due to the e-h exchange.

Supplementary Note 4. CW-PL valley polarization of another device (Device #3)

Here shows the results of gate-tunable PL polarization of Device #3 with excess energy of 29 meV. Supplementary Figure 3 (a) shows the polarized PL emission at different gate voltages. Similar to the results shown in the main text, the negligible DVP of excitons and trions in NCR (middle column in Supplementary Figure 3 (a)) gradually increases with increasing carrier density, to nearly 15 % and 30 % in the negatively and positively charged region, respectively, shown in Supplementary Figure 3 (b).

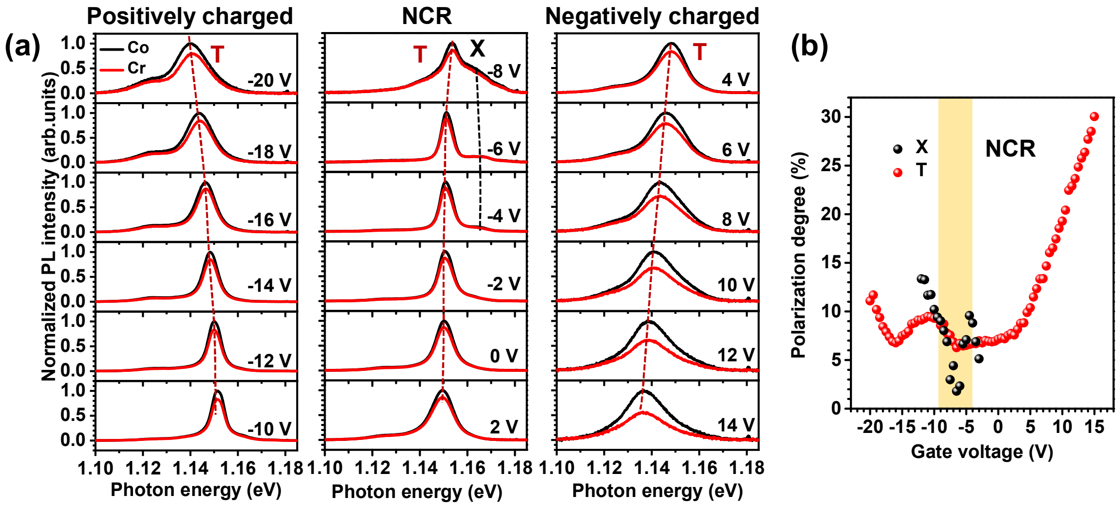

Supplementary Figure 3

Gate-tunable PL polarization of Device #3. (a) Polarized PL at different gate voltages. The black and red curves represent the same (Co) and opposite (Cr) circularly polarized emission with respect to pump. The PL peaks of trions and excitons are tracked by red and black dashed lines, respectively. (b) PL DVP as a function of gate voltages. Red and black dots denotes to trions and excitons, respectively. The neutrally charged region (NCR) is marked with yellow bar.

Supplementary Note 5. Determination of gate-controlled exciton and trion density

To correlate the PL with the estimated carrier densities, we calculate the pump-induced carrier densities in the CW-PL measurement. In this section, we use the steady-state solution of the rate equations that incorporate the mass-action law to determine the equilibrium densities of excitons and trions. Assuming that the optical pumping n_p only generates excitons n_X. Before pumping, the effective doping density n_D is determined by gating and the initial defect-induced electrons. In the presence of the optical pumping, part of the background electrons forms trions n_T by combining with photo-excited excitons. For simplicity, only the electron-trions are considered, thus we have $n_D = n_e + n_T$, where n_e is the free electrons left after the trion formation. These exciton-to-trion (X-T) coupled processes are described by the following rate equations:

$$\frac{dn_X}{dt} = g - n_X\Gamma_X^r - n_X\Gamma_{XT} + n_T\Gamma_{dis} \quad \text{(S1a)}$$

$$\frac{dn_T}{dt} = -n_T\Gamma_T^r + n_X\Gamma_{XT} - n_T\Gamma_{dis} \quad \text{(S1b)}$$

where Γ_X^r (Γ_T^r) is the recombination rate of excitons (trions) and Γ_{dis} is the dissociation rate of trions. To quantitatively analyze and compare the rates of each relaxation process, the X-T conversion term is written as $n_X\Gamma_{XT}$, where Γ_{XT} is defined as the conversion rate. The rate of X-T conversion is related to the free electron density (n_e). At cryogenic temperatures, the process of X-T conversion ($1/\Gamma_{XT}$) occurs within a few ps, while the dissociation time of trions ($1/\Gamma_{dis}$) is in the range of microsecond due to the relatively larger binding energies compared to conventional semiconductors, thus the dissociation process of trions into excitons is not taken into account in our discussion. The X-T coupled rate equations can be simplified as:

$$\frac{dn_X}{dt} = g - n_X\Gamma_X^r - n_X\Gamma_{XT} \quad \text{(S2a)}$$

$$\frac{dn_T}{dt} = -n_T\Gamma_T^r + n_X\Gamma_{XT} \quad \text{(S2b)}$$

In the CW measurement, the exciton generation rate $g = \frac{\eta P}{\hbar\omega S}$ is constant, and the carrier density induced by pumping is $n_p = g/\Gamma_X^r$. The density ratio of trions and excitons can be obtained from

the mass-action law (or the Saha ionization equation[7]: $\frac{n_{\mathrm{T}}}{n_{\mathrm{X}}}=\frac{n_{\mathrm{e}}}{K(T)}$, where $K(T)=\frac{4m_{\mathrm{e}}m_{\mathrm{X}}}{m_{\mathrm{T}}}\cdot\frac{k_B T}{\pi\hbar^2}\cdot\exp\left(-\frac{E_{\mathrm{T}}^{\mathrm{b}}}{k_B T}\right)$ is a temperature-dependent equilibrium constant. By solving equations S2a and Sb, we can obtain the following:

$$n_{\mathrm{p}}=n_{\mathrm{X}}+\frac{\Gamma_{\mathrm{T}}^{\mathrm{r}}}{\Gamma_{\mathrm{X}}^{\mathrm{r}}}n_{\mathrm{T}} \tag{S3a}$$

$$n_{\mathrm{T}}=n_{\mathrm{X}}\frac{\Gamma_{\mathrm{XT}}}{\Gamma_{\mathrm{T}}^{\mathrm{r}}}=n_{\mathrm{X}}\frac{n_{\mathrm{e}}}{K(T)} \tag{S3b}$$

Then the density of excitons and trions at given pump density n_p and charge density n_{D} can be calculated as

$$n_{\mathrm{X}}=n_{\mathrm{p}}-\frac{\Gamma_{\mathrm{T}}^{\mathrm{r}}}{\Gamma_{\mathrm{X}}^{\mathrm{r}}}n_{\mathrm{T}} \tag{S4a}$$

$$n_{\mathrm{T}}=\frac{n_{\mathrm{P}}+n_{\mathrm{D}}\frac{\Gamma_{\mathrm{T}}^{\mathrm{r}}}{\Gamma_{\mathrm{X}}^{\mathrm{r}}}+K(T)-\sqrt{\left(n_{\mathrm{P}}+n_{\mathrm{D}}\frac{\Gamma_{\mathrm{T}}^{\mathrm{r}}}{\Gamma_{\mathrm{X}}^{\mathrm{r}}}+K(T)\right)^2-4n_{\mathrm{P}}n_{\mathrm{D}}\frac{\Gamma_{\mathrm{T}}^{\mathrm{r}}}{\Gamma_{\mathrm{X}}^{\mathrm{r}}}}}{2\frac{\Gamma_{\mathrm{T}}^{\mathrm{r}}}{\Gamma_{\mathrm{X}}^{\mathrm{r}}}} \tag{S4b}$$

The photo-excited carrier density n_{P} is estimated to be ~ 10^{11} cm^{-2} for the pumping by a 633 nm HeNe laser. The electrically-gated background charge density n_{D} in our ML-$MoTe_2$ devices can be estimated from the gate-dependent measurement with a simplified model using Fermi energy $E_{\mathrm{F}}=\pi\hbar^2 n_{\mathrm{D}}/m_{\mathrm{e}}$, where m_{e} is the effective electron mass for $MoTe_2$ [5]. And the Fermi energy ($\mathrm{E_F}$) can be obtained from $E_{\mathrm{X}}-E_{\mathrm{T}}=E_{\mathrm{T}}^{\mathrm{b}}+E_{\mathrm{F}}$, where the energy splitting between exciton (E_{X}) and trion (E_{T}) resonances increases linearly with the increased charge density, the trion binding energy ($E_{\mathrm{T}}^{\mathrm{b}}$) is a constant value to be 19 meV for electron-trion ($E_{\mathrm{T-}}^{\mathrm{b}}$) and 22 meV for hole-trion ($E_{\mathrm{T+}}^{\mathrm{b}}$), determined from the energy splitting at neutrally charged region, as shown in the mapping of PL emission with gate voltages in the following SM Section S8. The n_{D} is calculated to be at 10^{11} ~ 10^{12} cm^{-2}. For simplicity, the ratio of the recombination rates of trions ($\Gamma_{\mathrm{T}}^{\mathrm{r}}=1/\tau_{\mathrm{T}}$) to excitons ($\Gamma_{\mathrm{X}}^{\mathrm{r}}=1/\tau_{\mathrm{X}}$) at different gate voltages is assumed to be a constant as, $\Gamma_{\mathrm{T}}^{\mathrm{r}}/\Gamma_{\mathrm{X}}^{\mathrm{r}}=0.1$, or $\tau_{\mathrm{X}}/\tau_{\mathrm{T}}=0.1$

on the same order of maginitude as what we measured by pump-probe experiments. The trion and exciton density are calculated based on the estimated carrier density n_D (gate-induced) and n_p (pump-induced) through equation S4a and S4b. The parameters used in the theoretical calculation are listed in Supplementary Table 1.

Supplementary Table 1

Definitions and values of parameters used in theoretical calculation

Parameter	Definition	Value
m_e	Electron mass	$0.69\ m_0$ [5]
m_h	Hole mass	$0.66\ m_0$ [5]
E^b_{T-}	Electron-trion binding energy	19 meV
E^b_{T+}	Hole-trion binding energy	22 meV
T	Temperature	10 K

Supplementary Note 6. Determination of DVP for background carriers

The background carriers can be polarized by circularly polarized excitation, which has been studied in W-based TMDCs with singlet and triplet trion species[8]. In $MoTe_2$, the singlet trion is dominant and the polarization of background carriers can be inferred from the PL polarization of excitons and trions. The polarization of background carriers is mainly caused by the formation and intervalley scattering processes of trions, as shown in Supplementary Figure 4. An intervalley scattering event of K/K'-valley trions through the simultaneous spin-flip of the three particles will lead to the switching of the background carriers from the K'/K valley to the K/K' valley. Since this type of spin-flip process for carriers is difficult to realize (experimentally verified by the long intervalley scattering time of trions in our system), the valley polarization of free carriers of this origin is weak. Therefore, the polarization of background carriers is dominantly influenced by the trion formation process.

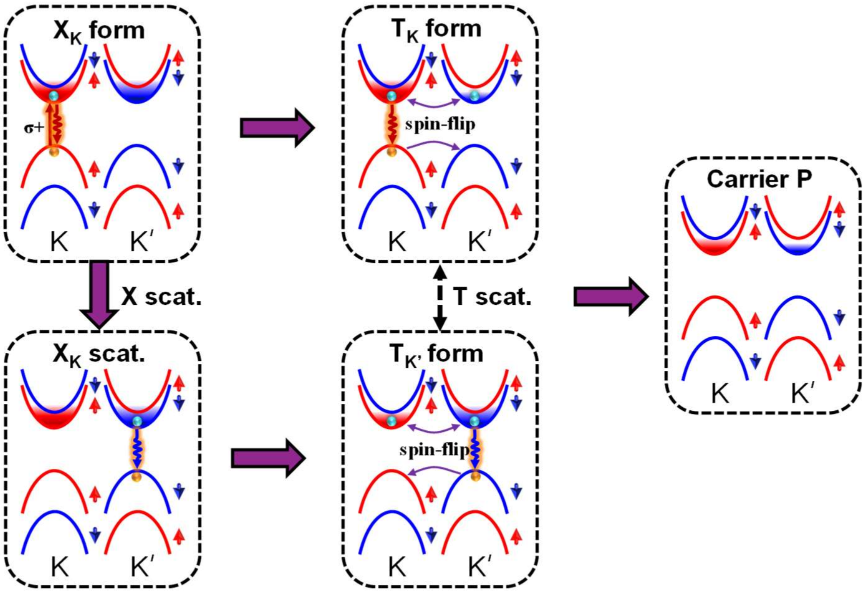

Supplementary Figure 4

Dynamical process of background-carrier polarization. Colored dots represent electrons or holes forming excitons or trions, while the red and blue shades represent "free" electrons in the two valleys. Formation of K-valley trion: A K-valley trion $\mathrm{T_K}$ consists of an exciton in K valley and an electron from the background carrier in K' valley. The intervalley scattering of the K-valley trion causes the simultaneous valley switching for both the electron and the exciton, leaving one less "free" electron in the K' valley and one more "free" electron in the K valley after trion recombination. Formation of K'-valley trion $\mathrm{T_{K'}}$: The intervalley scattered excitons in K' valley combine with background carrier in K valley. The intervalley scattering of the K'-valley trion will lead to the reduction of free carriers in K valley.

Supplementary Figure 4 shows the dynamical process of the carrier polarization. Initially, the total background carriers are equally distributed in K and K' valley. The formation of trions ($n_{\mathrm{T}}^{\mathrm{K/K'}}$) is the combination of excitons ($n_{\mathrm{X}}^{\mathrm{K/K'}}$) in the same valley and free carriers ($n_{\mathrm{e}}^{\mathrm{K'/K}}$) in the opposite valley. Based on the mass-action law, we have $n_{\mathrm{T}}^{\mathrm{K}} = \frac{n_{\mathrm{X}}^{\mathrm{K}} n_{\mathrm{e}}^{\mathrm{K'}}}{K(T)}$ and $n_{\mathrm{T}}^{\mathrm{K'}} = \frac{n_{\mathrm{X}}^{\mathrm{K'}} n_{\mathrm{e}}^{\mathrm{K}}}{K(T)}$, where $K(T) = \frac{4 m_{\mathrm{e}} m_{\mathrm{X}}}{m_{\mathrm{T}}} \cdot \frac{k_B T}{\pi \hbar^2} \cdot \exp\left(-\frac{E_{\mathrm{T}}^{\mathrm{b}}}{k_B T}\right)$ is an equilibrium constant. Thus the population of free carriers

in each valley is proportional to the population ratio of trions to excitons, as $n_e^{K/K'} \propto \frac{n_T^{K'/K}}{n_X^{K'/K}}$. The formation of K-valley trion will reduce the carrier population in K' valley. At the same time, the major portion of the cross-polarized emission from trions comes from the trion formation in the K' valley, due to the much stronger intervalley scattering of excitons. The formation process of K'-valley trions will reduce the free carriers in K valley. Therefore, the carrier polarization is related to the redistribution of excitons and trions in two valleys. The polarization degree of free carriers (P_e) can be written as

$$P_e = \frac{n_e^K - n_e^{K'}}{n_e^K + n_e^{K'}} \propto \frac{n_X^K n_T^{K'} - n_X^{K'} n_T^K}{n_X^K n_T^{K'} + n_X^{K'} n_T^K} \quad \text{(S5)}$$

The polarization degree of excitons (P_X) and trions (P_T) can be defined as

$$P_X = \frac{n_X^K - n_X^{K'}}{n_X^K + n_X^{K'}} \quad \text{(S6a)}$$

$$P_T = \frac{n_T^K - n_T^{K'}}{n_T^K + n_T^{K'}} \quad \text{(S6b)}$$

Combining the equations above we obtain that

$$P_e = \frac{P_X - P_T}{1 - P_T P_X} \quad \text{(S7)}$$

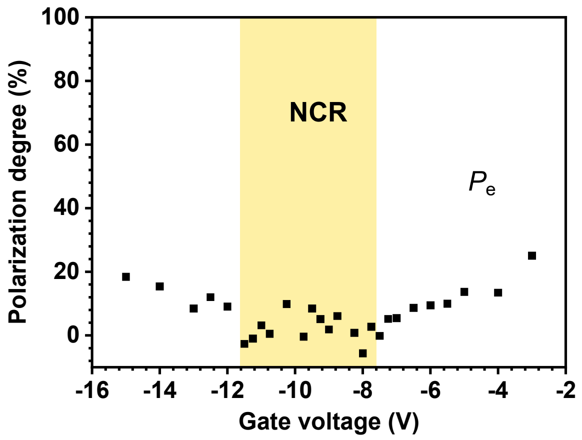

Supplementary Figure 5

Calculated polarization degrees of free carriers at different gate voltages. The neutrally charged region (NCR) is marked by yellow shaded area.

In the CW measurement, we can use the DVP for excitons and trions to obtain the value of P_e. Although the intervalley scattering time of both trions and free carriers can be long, the polarization degree P_e, which is influenced by the trion formation process, is calculated to be quite low. Supplementary Figure 5 shows the calculated P_e of Device #1 at different gate voltages. P_e is close to zero at the charge neutrality, and increases to less than 20 % with the increase of background carrier density. In theory, such weak polarization of background carriers could lead to a weak circular dichroism if free-carrier absorption is important as in the case of high density and for long wavelengths.

Supplementary Note 7. Possibility of trion formation in neutral ML-MoTe$_2$

The trion formation process considered in the main text is a bimolecular process whereby an exciton captures a charged carrier. This consideration is intuitively justified by the fact that the exciton binding energy (~ 580 meV)[9] is much larger than the trion binding energy (~20 meV). In this section, we consider the possibility of the direct trimolecular formation process[8,10] of trions in a neutral system. The density of exciton, electron-trion and hole-trion are related through the Saha equation as following:

$$\frac{n_e n_h}{n_X} = K_X(T) = \frac{g_e g_h}{g_X} \cdot \frac{m_e m_h}{m_X} \cdot \frac{k_B T}{2\pi\hbar^2} \cdot \exp\left(-\frac{E_{Xb}}{k_B T}\right) \tag{S8a}$$

$$\frac{n_X n_e}{n_{T2^-}} = K_2^-(T) = \frac{g_X g_e}{g_T} \cdot \frac{m_X m_e}{m_{T^-}} \cdot \frac{k_B T}{2\pi\hbar^2} \cdot \exp\left(-\frac{E_T^b}{k_B T}\right) \tag{S8b}$$

$$\frac{n_X n_h}{n_{T2^+}} = K_2^+(T) = \frac{g_X g_h}{g_T} \cdot \frac{m_X m_h}{m_{T^+}} \cdot \frac{k_B T}{2\pi\hbar^2} \cdot \exp\left(-\frac{E_T^b}{k_B T}\right) \tag{S8c}$$

$$\frac{n_e^2 n_h}{n_{T3^-}} = K_3^-(T) = \frac{g_e^2 g_h}{g_T} \cdot \frac{m_e^2 m_h}{m_{T^-}} \cdot \left(\frac{k_B T}{2\pi\hbar^2}\right)^2 \cdot \exp\left(-\frac{E_{Tb}}{k_B T}\right) \tag{S8d}$$

$$\frac{n_e n_h^2}{n_{T3^+}} = K_3^+(T) = \frac{g_e g_h^2}{g_T} \cdot \frac{m_e m_h^2}{m_{T^+}} \cdot \left(\frac{k_B T}{2\pi\hbar^2}\right)^2 \cdot \exp\left(-\frac{E_{Tb}}{k_B T}\right) \tag{S8e}$$

where n_e and n_h represent the electron and hole densities induced by optical pumping. In the absence of excess carriers, n_X is the exciton density. n_{T2^-} (n_{T2^+}) and n_{T3^-} (n_{T3^+}) denote the

densities of electron-trions (hole-trions) formed through the bimolecular and trimolecular process, respectively. $K(T)$, $K_2^{\pm}(T)$ and $K_3^{\pm}(T)$ are the equilibrium coefficients for exciton, bimolecular trion and trimolecular trion, respectively. The factor $g_{\mathrm{e,h,X,T}}=2$ is the spin degeneracy of the electron, hole, exciton and trion in the non-degenerate regime. E_{Xb} ($E_{\mathrm{Xb}}=\mu_{\mathrm{X}}-(\mu_{\mathrm{e}}+\mu_{\mathrm{h}})$) and E_{Tb} ($E_{\mathrm{Tb}}=\mu_{\mathrm{T}}-(2\mu_{\mathrm{e}}+\mu_{\mathrm{h}})$) is defined as minus the binding energy of exciton and trion from free carriers, where $\mu_{\mathrm{e,h,X,T}}$ is the chemical potential. $E_{\mathrm{T}}^{\mathrm{b}}=E_{\mathrm{Tb}}-E_{\mathrm{Xb}}$ is the binding energy of trions formed from exciton with a free carrier. The ratio between exciton and trion density can be obtained through:

$$\frac{n_{\mathrm{X}}^3}{n_{\mathrm{T3^+}}n_{\mathrm{T3^-}}}=\frac{g_{\mathrm{X}}^3}{g_{\mathrm{T}}^2}\frac{m_{\mathrm{X}}^3}{m_{\mathrm{T+}}m_{\mathrm{T-}}}(\frac{k_BT}{2\pi\hbar^2})\exp\left(-\frac{2E_{\mathrm{T}}^{\mathrm{b}}-E_{\mathrm{Xb}}}{k_BT}\right) \tag{S9}$$

For the ML-MoTe2 system, E_{Xb} is ~ 580 meV, and E_T^b is ~ 20 meV. At low temperatures of 10 K, the value of the exponential factor $\exp(-\frac{2E_{\mathrm{T}}^{\mathrm{b}}-E_{\mathrm{Xb}}}{k_BT})$ in Eq. S9 is calculated to be $\exp(627)$, which is extremely large, implying the dominant formation of excitons rather than trions from photo-generated carriers in a charge-neutral system.

In addition, due to charge conservation, we have $n_{\mathrm{e}}=n_{\mathrm{h}}=N-n_{\mathrm{X}}=N_{\mathrm{ex}}$, in which N is the total density of absorbed photons. For simplicity, here we consider the situation of electron-trion only. Equations S8a, S8b and S8d can be changed to the following by variable substitution:

$$\frac{N_{\mathrm{ex}}^2}{(N-N_{\mathrm{ex}})}=K_{\mathrm{X}}(T)\propto\exp\left(-\frac{E_{\mathrm{Xb}}}{k_BT}\right) \tag{S10a}$$

$$\frac{(N-N_{\mathrm{ex}})N_{\mathrm{ex}}}{n_{\mathrm{T2^-}}}=K_2^-(T)\propto\exp\left(-\frac{E_{\mathrm{T}}^{\mathrm{b}}}{k_BT}\right) \tag{S10b}$$

$$\frac{N_{\mathrm{ex}}^3}{n_{\mathrm{T3^-}}}=K_3^-(T)\propto\exp\left(-\frac{E_{\mathrm{Tb}}}{k_BT}\right) \tag{S10c}$$

Apparently, for low excitation intensity with moderate N, the exciton density varies as $\mathrm{O}(N^2)$, the trion density through trimolecular process varies as $\mathrm{O}(N_{\mathrm{ex}}^3)$. The trion density $n_{\mathrm{T3^-}}$ is highly

dependent on the fraction of the total absorbed photon density in the free carrier states. The trimolecular formation process of trions need to be taken into account either at sufficiently high doping densities or pump densities close to the Mott transition with large portion of free carriers. In our experiment, the background carrier density is estimated to be $10^{11} \sim 10^{12}$ cm^{-2}. And the excitation level is at ~ 10^{11} cm^{-2} in the CW experiments and at ~ 10^{12} cm^{-2} in the pump-probe experiments. Therefore, trions are predominantly formed from the photo-generated excitons through the capture of an electron or hole in a doped system.

Supplementary Note 8. Phenomenological modeling of valley-resolved rate equations

In order to understand the gate-dependent valley dynamics, we analyze the optical processes occurred in the sample after optical pumping, as depicted in Fig. 2 (A) in the main text. After being excited by right-circularly polarized light, e-h pairs are excited in K valley to form excitons due to the optical selection rules in TMDC MLs. Then the excitons will either recombine to emit PL or convert to trions with excess carriers induced by electrical gating. Due to the ultrafast intervalley scattering process, excitons and trions can also redistribute to K' valley, leading to the reduction of DVP.

As mentioned in the main text, the exciton and trion signals can be analyzed by valley-resolved rate equations, as

$$\frac{dn_{\mathrm{X}}^{\mathrm{K/K'}}}{dt} = g^{\mathrm{K/K'}} - n_{\mathrm{X}}^{\mathrm{K/K'}}\Gamma_X^r - n_{\mathrm{X}}^{\mathrm{K/K'}}\Gamma_{\mathrm{XT}} + (n_{\mathrm{X}}^{\mathrm{K'/K}} - n_{\mathrm{X}}^{\mathrm{K/K'}})\Gamma_{\mathrm{X}}^{\mathrm{sk}} \tag{S11a}$$

$$\frac{dn_{\mathrm{T}}^{\mathrm{K/K'}}}{dt} = -n_{\mathrm{T}}^{\mathrm{K/K'}}\Gamma_{\mathrm{T}}^{\mathrm{r}} + n_{\mathrm{X}}^{\mathrm{K/K'}}\Gamma_{\mathrm{XT}} + (n_{\mathrm{T}}^{\mathrm{K'/K}} - n_{\mathrm{T}}^{\mathrm{K/K'}})\Gamma_{\mathrm{T}}^{\mathrm{sk}} \tag{S11b}$$

where $g^{\mathrm{K/K'}}$ is the generation rate of excitons, which is determined by the circularly-polarized pump (Gaussian) pulses. The $n_{\mathrm{X}}^{\mathrm{K/K'}}$ ($n_{\mathrm{T}}^{\mathrm{K/K'}}$) stands for the population of excitons (trions) in K/K' valley, $\Gamma_{\mathrm{X}}^{\mathrm{r}}(\Gamma_{\mathrm{T}}^{\mathrm{r}})$ and $\Gamma_{\mathrm{X}}^{\mathrm{sk}}(\Gamma_{\mathrm{T}}^{\mathrm{sk}})$ denote the recombination rate and intervalley scattering rate of excitons (trions), respectively. The intervalley scattering rate is determined by which mechanisms of depolarization dominates in the system, fast e-h exchange for excitons, and the relatively slow

spin-flip scattering for trions. The conversion rate from excitons to trions is defined as Γ_{XT}. The dissociation of trions into excitons is not taken into account. At cryogenic temperatures, the exciton-to-trion (X-T) conversion occurs within a few picoseconds (ps), while the dissociation time of trions is in the range of microsecond due to the relatively larger binding energies compared to conventional semiconductors, thus can be neglected in our discussion.

Here we display the time-resolved solution of the rate equations to provide a general understanding of the decay process of carriers. Considering the convolution of the Gaussian pulsed excitation, the population evolution of excitons and trions in K/K' valley can be expressed in the form of

$$n_{\mathrm{X}}^{\mathrm{K/K'}} = \sum_{i=1}^{2} C_{\mathrm{X}i} \cdot e^{-\Gamma_i t} \cdot \mathrm{erf}\left(\frac{t}{w} - \frac{w\Gamma_i}{2}\right) \quad \text{(S12a)}$$

$$n_{\mathrm{T}}^{\mathrm{K/K'}} = \sum_{i=1}^{4} C_{\mathrm{T}i} \cdot e^{-\Gamma_i t} \cdot \mathrm{erf}\left(\frac{t}{w} - \frac{w\Gamma_i}{2}\right) \quad \text{(S12b)}$$

where t is the delay time between pump and probe pulses. The erf () stands for the error function, and w is the cross correlation width, which is determined by the pulse width of the Gaussian pulse, as $\mathrm{w} = \frac{FWHM}{2\sqrt{2\ln(2)}} = \frac{0.5ps}{2.355}$. C_i and Γ_i is the relative amplitudes and decay rates of each optical processes. The detailed expression of each decay rate is listed in

Supplementary Table **2**. Here we can classify the decay channels into two types: intravalley decay and intervalley decay. For excitons, the two interconnecting decay channels, mainly influenced by intravalley decay $\Gamma_{\mathrm{X}}^{\mathrm{ia}}$ (including recombination and X-T conversion) and intervalley decay $\Gamma_{\mathrm{X}}^{\mathrm{ir}}$ (including intervalley decay) of excitons. While for trions, due to the X-T interactions, besides the terms for excitons, there exist another two relaxation channels, influenced by intravalley decay $\Gamma_{\mathrm{T}}^{\mathrm{ia}}$ (mainly by recombination) and intervalley decay $\Gamma_{\mathrm{T}}^{\mathrm{ir}}$ (including intervalley scattering) of trions.

Supplementary Table 2

Relaxation rate derived from valley-resolved rate equations

Relaxation rate	Detailed expression	Decay channel
Γ_1	$\Gamma_X^r + \Gamma_{XT}$	Γ_X^{ia}
Γ_2	$\Gamma_X^r + \Gamma_{XT} + 2\Gamma_X^{sk}$	$\Gamma_X^{ia} + \Gamma_X^{ir}$
Γ_3	Γ_T^r	Γ_T^{ia}
Γ_4	$\Gamma_T^r + 2\Gamma_T^{sk}$	$\Gamma_T^{ia} + \Gamma_T^{ir}$

After the excitation pulse, the valley-resolved dynamics of excitons and trions can be fitted by exponential functions, as shown in Fig. 3 and Fig. 4 in the main text. The aforementioned time constants can be extracted by fitting the rate equations. The intravalley and intervalley decay process of excitons and trions are discussed separately in the following parts.

i) Intravalley decay rate of excitons and trions

The sum of populations in K and K' valley (Equation (S12a) and (S12b)) will cancel the terms of intervalley scattering, thus reflecting the intravalley decay time constants of excitons and trions. When the probe delay t is larger than the pulse width of excitation w, the exciton and trion population can be simplified as

$$n_X = C_{X1} \cdot e^{-\Gamma_X^{ia} t} \quad \text{(S13a)}$$

$$n_T = C_{T1} \cdot e^{-\Gamma_X^{ia} t} + C_{T3} \cdot e^{-\Gamma_T^{ia} t} \quad \text{(S13b)}$$

Supplementary Figure 6 (a) shows the transient differential reflectance spectra of excitons mapping with gate voltages. The signal intensity of excitons is stronger in NCR, indicating the preference of conversion to trions at higher charge density. The intravalley decay rate of excitons is determined by both the recombination Γ_X^r and X-T conversion rate Γ_{XT}. Since the effective decay rate is determined by the fastest relaxation process, thus is on the same time scale as the X-T conversion, within a few ps. However, the transient differential reflectance signal of excitons can be fitted by a bi-exponential function. The evolution dynamics thus can be divided into two stages, as shown in the colored area in Supplementary Figure 6 (b). The fast component in stage I

(purple area), is attributed to the decay of free excitons, while the slow component in stage II (blue area), is likely due to the existence of localized excitons induced by imperfections during sample preparation and defects. Supplementary Figure 6 (b) & (c) depicted the detailed differential reflectance curves at each gate voltages and the fitted decay time of free excitons (t_{X1}) and localized excitons (t_{X2}). The decay time of both the free excitons and localized excitons decreases at higher charge density. The decrease of free exciton decay time agrees well with the increasing probabilities of X-T conversion with increasing free carriers.

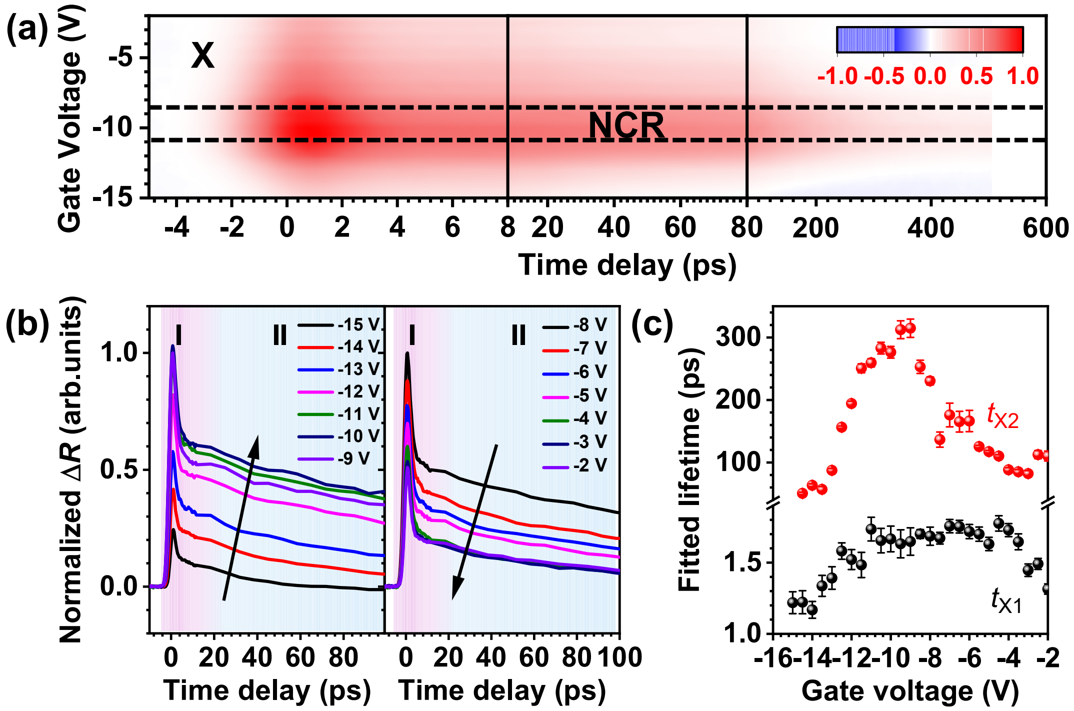

Supplementary Figure 6

Gate tuning of the evolution dynamics of excitons. The time-resolved dynamics of excitons at different gate voltages in the Device #3, same sample in Fig. 2 (c & d) in the main text. (a): the colored contour of differential reflectance decay of excitons mapped with gate voltages, with NCR marked by black dashed lines; (b): the decay curves as a function of gate voltages. (c): the fitted intravalley decay times with error bars at different gate voltages.

Supplementary Figure 7 show the same data set for trions. The transient differential reflectance spectra of trions mapping with gate voltages is shown in Supplementary Figure 7 (a). Similarly, as

shown in Supplementary Figure 7 (b), the evolution dynamics of trions can be divided into the two stages, which are related to the trion formation and trion recombination, respectively. In the stage I, almost the same time scale of free exciton decay, the transient signal of trions rapidly declines to negative values, which corresponds to the trion formation process. The trion formation time in ML-$MoTe_2$, as denoted as t_{T1} in Supplementary Figure 7 (c), is fitted to be in the single ps range, with a similar time constant and voltage dependence with free exciton decay time t_{X1} shown in Supplementary Figure 6 (c).

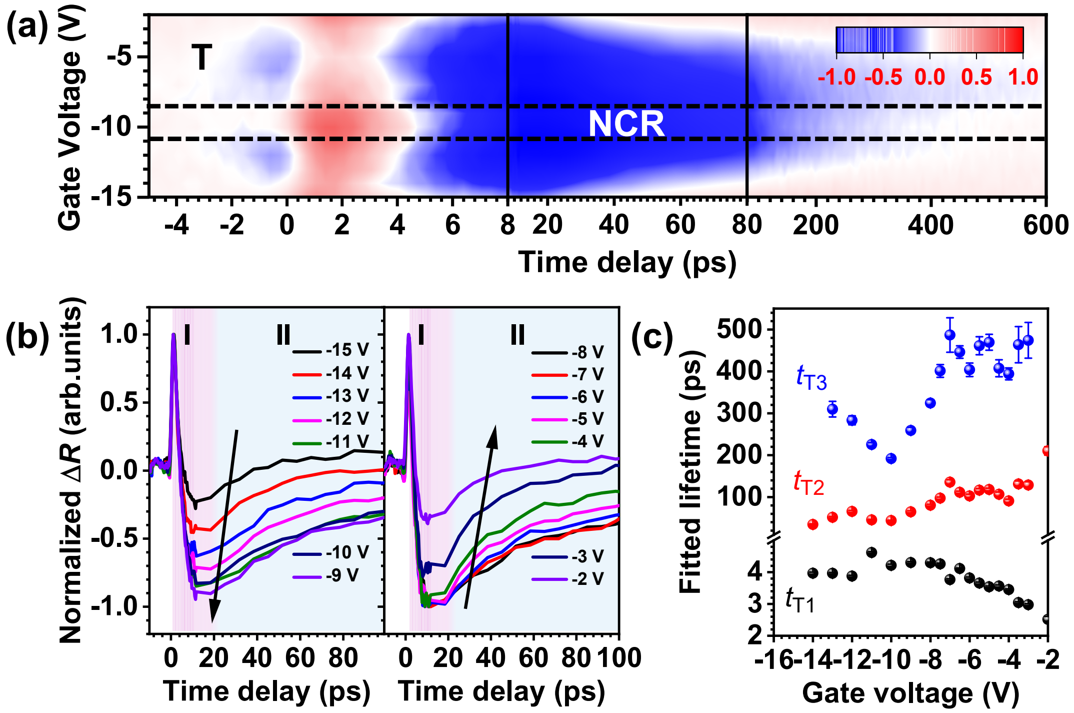

Supplementary Figure 7

Gate tuning of the evolution dynamics of trions. The time-resolved dynamics of trions at different gate voltages in the Device #3, same sample in Fig. 2 (c & d) in the main text. (a): the colored contour of differential reflectance decay of trions mapped with gate voltages, with NCR marked by black dashed lines; (b): the decay curves as a function of gate voltages. (c): the fitted intravalley decay times with error bars at different gate voltages.

The trion decay dynamics can be fitted by a bi-exponential function in this sample. The two time components may originate from the recombination process of mobile and immobile trion species

in the sample, which shows different behaviors with gating. As shown in Supplementary Figure 7 (c), at NCR, the decay time t_{T2} and t_{T3} both show a slight increase with the increased charge density. This is because the electrons left behind by the trion recombination have difficulties to find an empty state in the conduction band. At high charge density deviating from NCR, the long component t_{T3} increases in a wider range of gate voltages, which may related to the immobile trion state [11]. With further increasing background charges, the recombination rate of trions gets faster, due to the enhanced carrier interactions encouraged by the background population.

ii) Intervalley decay rate of excitons and trions

The intervalley decay rate can also be derived from the solutions of the valley-resolved rate equations. The DVP for excitons and trions can be calculated using the time-resolved solutions of exciton and trion population in each valley.

For excitons, the time-dependent exciton polarization can be derived as an exponential decay closely related to the intervalley decay rate of excitons (Γ_X^{ir}).

$$P_X = \frac{n_X^K - n_X^{K'}}{n_X^K + n_X^{K'}} = \frac{C_{X2}e^{-\Gamma_2 t}}{C_{X1}e^{-\Gamma_1 t}} = \frac{C_{X2}e^{-(\Gamma_X^{ia}+\Gamma_X^{ir})t}}{C_{X1}e^{-\Gamma_X^{ia}t}} \approx Ae^{-\Gamma_X^{ir}t} \quad \text{(S14)}$$

For trions, the DVP can be expressed as

$$P_T = \frac{n_T^K - n_T^{K'}}{n_T^K + n_T^{K'}} = \frac{C_{T4}e^{-\Gamma_4 t} + C_{T2}e^{-\Gamma_2 t}}{C_{T3}e^{-\Gamma_3 t} + C_{T1}e^{-\Gamma_1 t}} = \frac{C_{T4}e^{-(\Gamma_T^{ia}+\Gamma_T^{ir})t} + C_{T2}e^{-(\Gamma_X^{ia}+\Gamma_X^{ir})t}}{C_{T3}e^{-\Gamma_T^{ia}t} + C_{T1}e^{-\Gamma_X^{ia}t}} \quad \text{(S15)}$$

The trion polarization decay is related to the intervalley scattering of both excitons and trions. After the decay of free excitons, the time-dependent trion polarization can be simplified as

$$P_T = \frac{n_T^K - n_T^{K'}}{n_T^K + n_T^{K'}} = \frac{C_{T1}e^{-(\Gamma_T^{ia}+\Gamma_T^{ir})t}}{C_{T2}e^{-\Gamma_T^{ia}t}} \approx Be^{-\Gamma_T^{ir}t} \quad \text{(S16)}$$

At stage II of the trion evolution, as illustrated in Supplementary Figure 7 (b), the trion polarization decays exponentially with the decay rate of Γ_T^{ir}. Thus by exponential fitting, the intervalley decay rate of excitons and trions can be obtained directly from the time-resolved polarization decay. The extracted intervalley decay time $1/\Gamma_X^{ir}=1/2\Gamma_X^{sk}$ and $1/\Gamma_T^{ir}=1/2\Gamma_T^{sk}$ are shown in Fig. 3b and Fig. 4b in the main text.

Supplementary Note 9. Steady-state optical characterization

To investigate the mutual conversions of the excitonic complexes in ML-$MoTe_2$, steady-state PL and absorption measurements were conducted at different gate voltages. The absorption features can be derived from differential reflectance, defined as: $-\alpha(0) \propto (R_S - R_0)/R_0$, where $R_S(R_0)$ represents the reflectance with (without) $MoTe_2$.

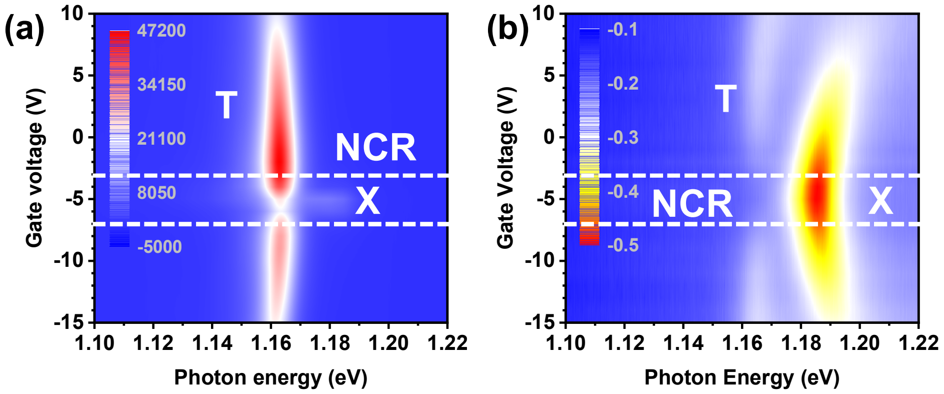

Supplementary Figure 8

Gate tuning X-T interactions in ML-$MoTe_2$. PL (a) and absorption (b) spectra mapping as a function of gate voltages in the Device #4. The white dashed lines indicate the NCR.

As can be seen from both PL and absorption spectra in Device #4 shown in Supplementary Figure 8, the intensity and spectral positions of excitons (X) and trions (T) can be effectively controlled by gating. Trion intensity reaches a minimum value at negative voltages, denoted by NCR, where exciton features are observable, indicating the sample is intrinsically negatively charged. Both PL and absorption spectra vary symmetrically with respect to NCR. As the gate voltage increases deviating from NCR, the conversion of excitons to trions is encouraged with the increasing densities of electrons or holes. Both PL and absorption of trions increases with gate voltages, and exciton intensity gradually diminishes at high gate voltages.

Supplementary Note 10. Spectra fitting of time-resolved differential reflectance

The exciton and trion resonances can be first determined in the CW reflectance measurement by the Gaussian fitting, as shown in the top panel in Supplementary Figure 9. The bottom panel in

Supplementary Figure 9 shows the fitted results for the transient reflectance $\Delta R = R_p - R_0$ at $\Delta t = 5$ ps. Based on the resonance energies of exciton (X) and trion (T) in the CW measurement, the transient reflectance in the pump-probe measurement can be well fitted with four peaks, representing without pump (X_0, T_0) and with pump (X_p, T_p). The pump-induced energy shifts can be read directly from the difference between the lines of CW and pump-probe spectra in Supplementary Figure 9, which is ~ 1.9 meV and ~ 0.3 meV for excitons and trions, respectively. In our pump-probe measurement, the linewidth of pump and probe pulses were spectrally filtered to be ~ 3 meV. Therefore, the determination of valley lifetime is not affected by the variation of pump densities in the CW and pump-probe experiments.

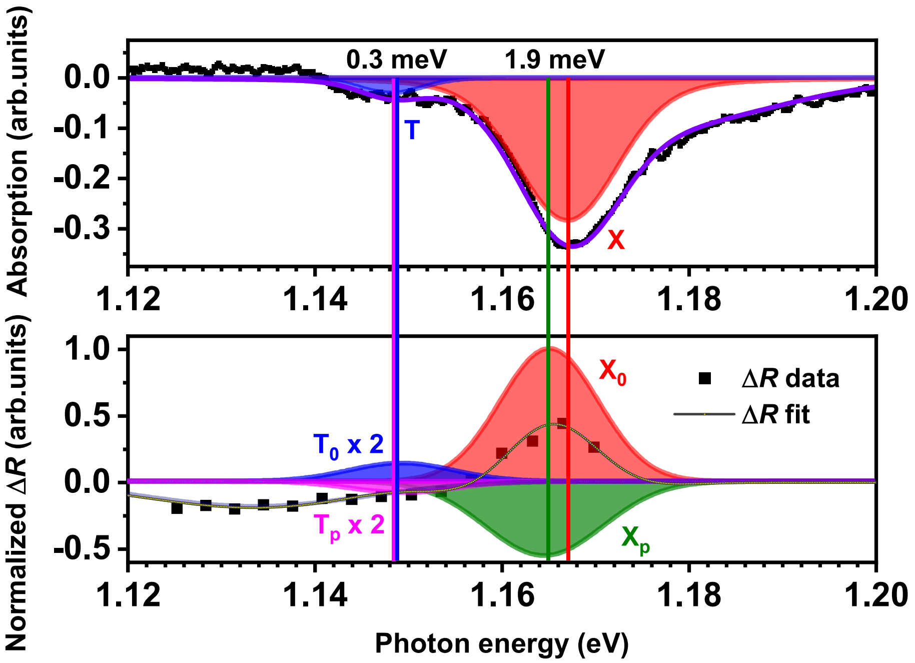

Supplementary Figure 9

Fitting results of differential reflectance spectra. The red and blue Gaussian peaks represent exciton (X) and trion (T) absorptions before pump, respectively, labelled as X and T in the CW (top) and X_0 and T_0 in the transient differential reflectance (bottom) experiments. The measured $\Delta R = R_p - R_0$ spectrum (bottom) is fitted with multiple Gaussian peaks. The dots represent the raw data measured at a time delay Δt of 5 ps, and the gray line represents the sum of the fitted peaks. X_p and T_p represent the resonance energies after the pump and are marked with the green and magenta lines respectively.

Supplementary Note 11. Pump induced carrier density in time-resolved pump-probe measurements

In pump-probe measurements, we use a 1040 nm femtosecond laser, with repetition rate of 400 kHz. The pump energy was tuned to be near-resonant to exciton energy at ~ 1.18 eV, with an excess energy (ΔE) of ~ 10 meV. The pump fluence of pump pulses can be calculated to be 20 $\mu J \cdot cm^{-2}$. The absorption at pump energy is extracted from the PLE measurement of ML-$MoTe_2$ [12], the absorption at near resonant excitation ($\Delta E \sim 10$ meV) is 3 %. The pump induced carrier density n_p is estimated to be 3×10^{12} cm^{-2}.

Supplementary Note 12. Pump (probe) energy dependent transient absorption spectroscopy

To better understand the carrier behaviors after pumping in ML-$MoTe_2$, the exciton and trion dynamics is detailed studied by tuning pump (probe) energies to exciton (trion) resonances, respectively, as shown in Supplementary Figure 10. The notations in the subfigures represent pump - probe energy, respectively. All the transient signals show a positive peak in the first few ps, close to the time resolution of our system. This peak can be attributed to the bleaching of absorption induced by pumping. As shown in the insets of Supplementary Figure 10 (a) & (b), the faster rise of T-X signal compared to X-X reflect the immediate coherent coupling of excitons and trions, similar to other works [13]. As has been discussed in the main text, the subsequent positive signal of excitons (X-X & T-X) originates from the band-filling effect, while the absorption of trions (T-T & X-T) changes to negative value caused by bandgap renormalization.

The exciton-trion coupling can be revealed by probing at exciton (trion) resonance and pumping at trion (exciton) resonance, denoted as T-X (X-T), shown in Supplementary Figure 10 (b) & (d), respectively. In Supplementary Figure 10 (b), the T-X signal shows an indistinguishable dip at time-zero point, which is likely caused by the optical processes related to higher order excitonic states. In Supplementary Figure 10 (d), the relatively weak positive peak in X-T compared to that in T-T is consistent with the fact that the reduced pumping efficiency at larger excess energy to trion resonance.

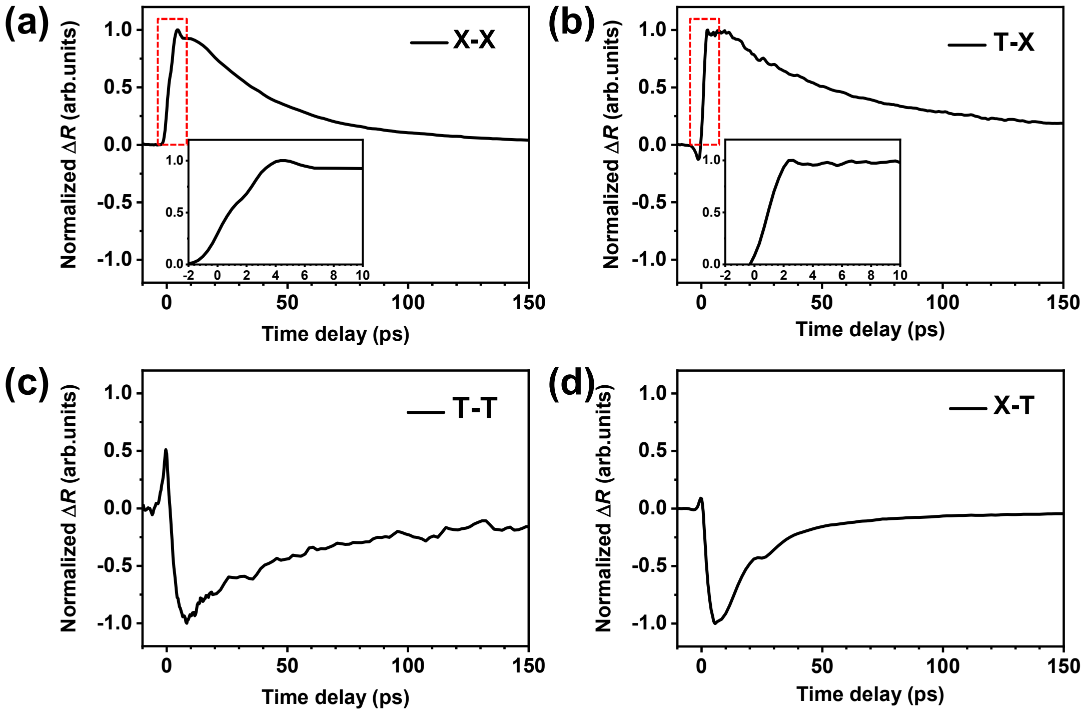

Supplementary Figure 10

Exciton and trion evolutions revealed by transient differential reflectance spectroscopy. (a - d) The exciton and trion dynamics with different combinations of pump-probe energies. The notations in the figure represent pump - probe energy, respectively. The inset of X-X and T-X spectrum shows the zoom-in view of exciton differential reflectance signal in the dashed frames.

Supplementary Note 13. Detailed electrical tuning of valley polarization dynamics

The detailed electrical tuning of valley dynamics is shown in Supplementary Figure 11 and

Supplementary Figure **12**. The X-T conversion is encouraged with increased carrier density and competes with the intervalley scattering of excitons, leading to the increase of exciton DVP.

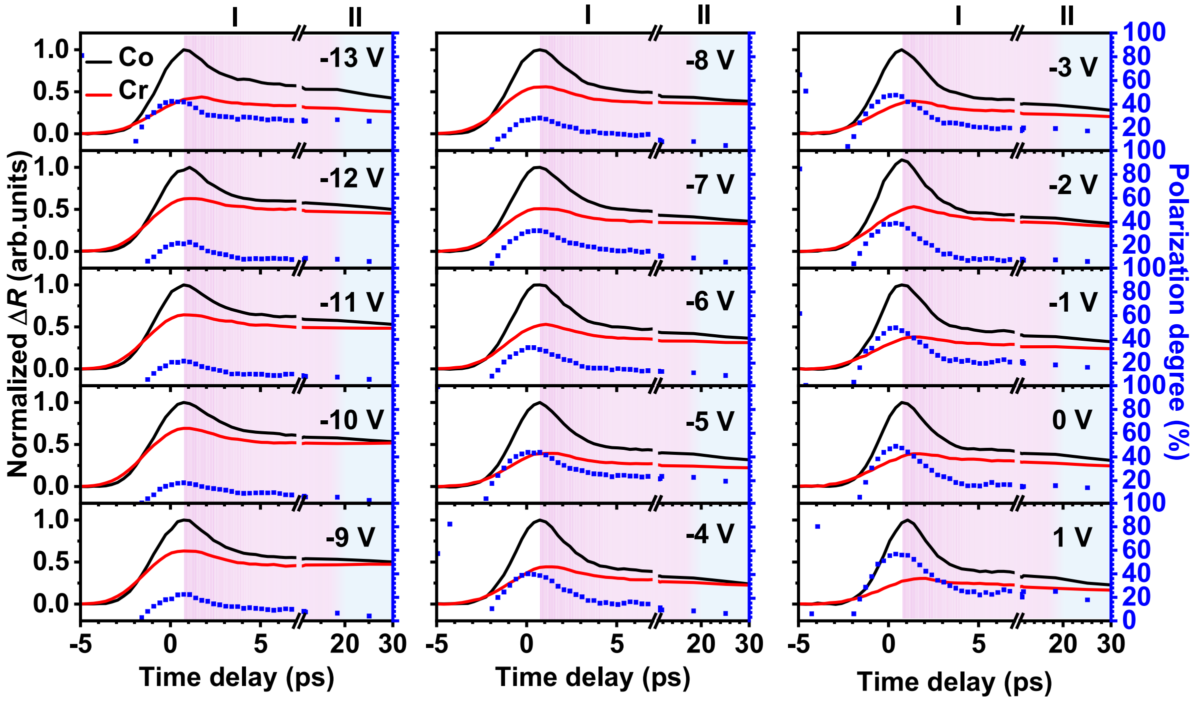

Supplementary Figure 11

Exciton valley dynamics resolved at different gate voltages. The exciton dynamics of Device #3, the same sample as in Fig. 3 in the main text. The exciton polarization persists longer at higher charge density region with the gate voltage below -13 V and above -7 V.

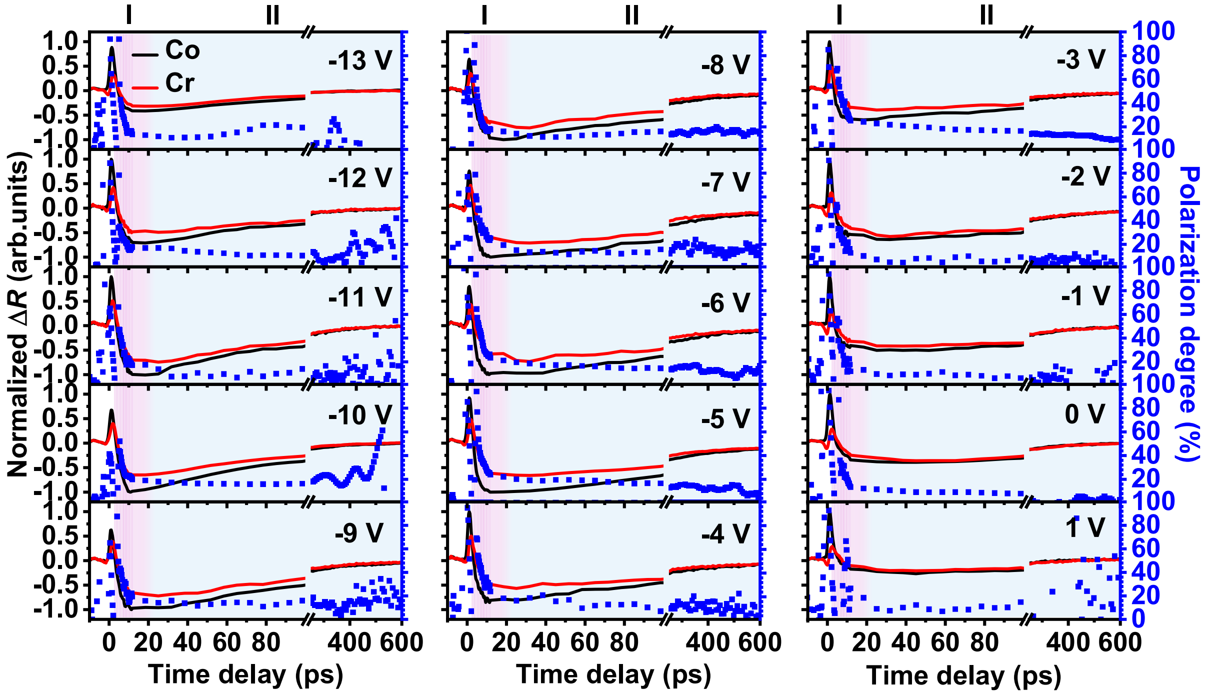

Supplementary Figure 12

Trion valley dynamics resolved at different gate voltages. The corresponding trion valley dynamics in Device #3, the same device as the one shown in Fig. 3 in the main text. This sample shows longer scattering time but lower DVP value than the Device #2 shown in Fig. 4 in the main text. The trion DVP can be maintained over 10 % at ~ 1000 ps at the NCR with gate voltage from -6 V to -9 V. While at higher charge density region, the trion DVP maintenance time is reduced due to the increased spin relaxation rate.

Supplementary Note 14. Influence of photo-induced carrier density on the intervalley scattering

The intervalley scattering process is in general affected by the photo-generated carrier densities. The valley dynamics of both excitons and trions have been studied at different carrier densities. Supplementary Figure 13 (a) and (b) show the exciton DVP dynamics for device #5 at ~ 6 × 10^{12} cm^{-2} and ~ 6 × 10^{13} cm^{-2}, respectively. The initial DVP at 0 ps is ~ 50 % at ~ 6 × 10^{12} cm^{-2} and increases to ~ 80 % at ~ 6 × 10^{13} cm^{-2}. However, the exciton intervalley relaxation time does not vary much with carrier density. The intervalley decay time of excitons is 0.93 ± 0.04 ps at ~ 6 ×

10^{12} cm^{-2} and 0.79 ± 0.03 ps at ~ 6 × 10^{13} cm^{-2}. Supplementary Figure 13 (c) and (d) show the trion DVP dynamics for device #6. The intervalley scattering time can not be well fitted with exponential functions. As can be seen, the intervalley relaxation time of trions does not vary much with pump-induced carrier density. The trion DVP at both ~ 1.2 × 10^{13} cm^{-2} and ~ 2.4 × 10^{14} cm^{-2} can maintain valley polarized over 100 ps.

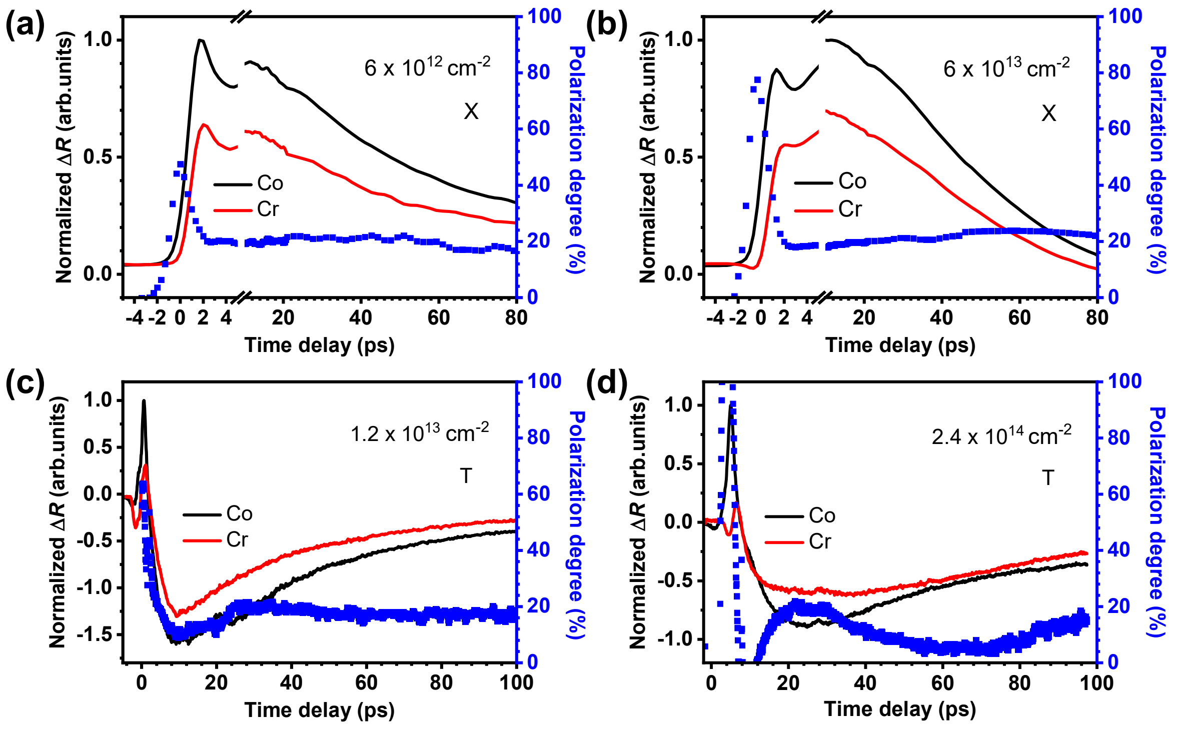

Supplementary Figure 13

Helicity-resolved reflectance and time-resolved DVP of excitons and trions at different pump-induced carrier density. Exciton valley dynamics in Device #5 at carrier density of ~ 6 × 10^{12} cm^{-2} (a) and ~ 6 × 10^{13} cm^{-2} (b); Trion valley dynamics in Device #6 at pump fluence of ~ 1.2 × 10^{13} cm^{-2} (c) and ~ 2.4 × 10^{14} cm^{-2} (d). The black and red curves indicate the differential reflectance with the same (Co) and opposite (Cr) polarization with pump. The blue dots represent the DVP at different time delay.

<u>**Supplementary Note 15. Fitting reliability of the valley dynamics**</u>

The intervalley decay time of trions is obtained by linearly fitting the polarization decay in natural logarithmic scale for accuracy. Supplementary Figure 14 (a) & (b) show the representative fitting results of the trion intervalley decay time shown in Fig. 4 in the main text. Supplementary Figure 14 (c &d) shows the representative fitting results of trion intervalley decay time of Device #3.

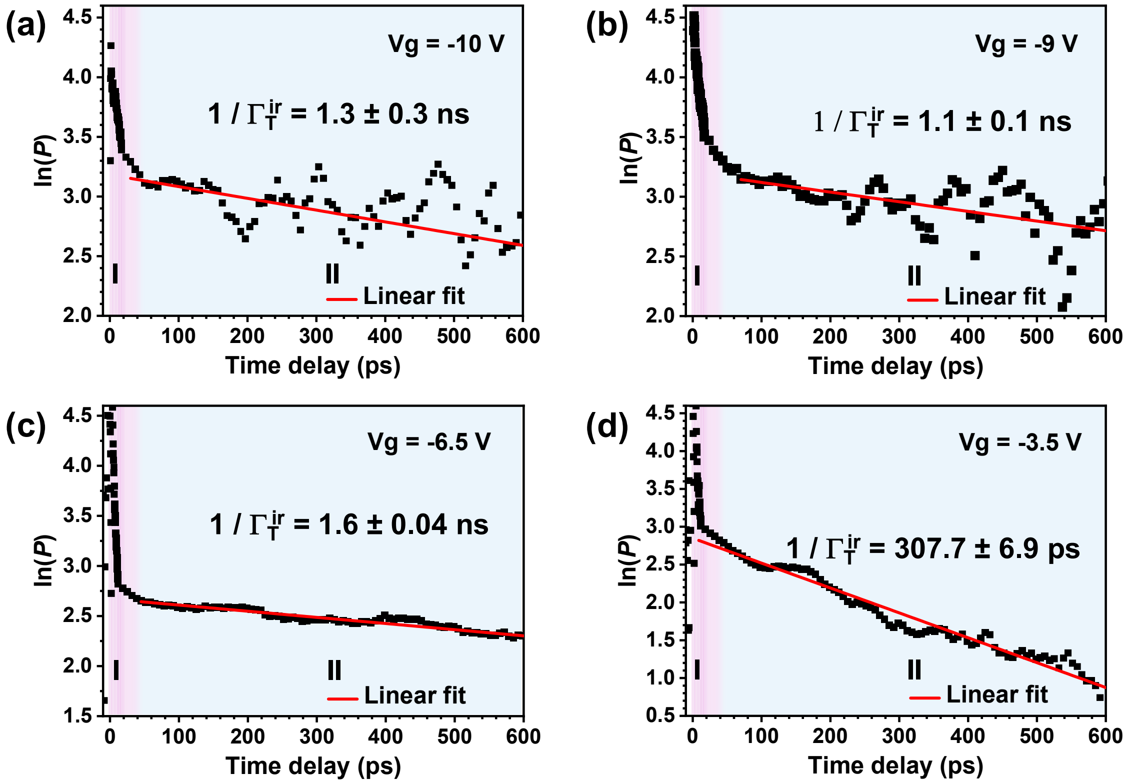

Supplementary Figure 14

Representative fitting results of trion polarization in natural logarithmic scale. The trion polarization decay of Device #2 (a & b) and Device #3 (c & d) is fitted. The black dots denote the experiment data of trion polarization in natural logarithmic scale, and the red solid lines indicate the linear fit with slope of $\Gamma_T^{sk} = \Gamma_T^{ir} / 2$. The corresponding fitted intervalley decay time $1/\Gamma_T^{ir}$ is labelled. Device #2 is the same device in Fig. 4 in the main text, and Device #3 is the same device in Fig.3 in the main text.

Supplementary Note 16. Calculation of spin relaxation time by DP and BAP mechanism

The ultra-long valley scattering of trions originate from the difficulties of the spin-flip of a single particle. The spin relaxation of free carriers in semiconductor can be mainly attributed to four mechanisms, the Elliot-Yafet (EY) mechanism, the Dyakonov-Perel (DP) mechanism, the Bir-Aronov-Pikus (BAP) mechanism, and the hyperfine interaction [14]. The hyperfine interaction is always neglected in 2D TMDC. And the EY mechanism is also negligible due to the marginal in-plane spin mixing in TMDC materials. So here we focus on the DP and BAP mechanisms accounting for the intervalley electron and hole spin relaxation processes, respectively.

i) DP mechanism

The spin-orbit coupling (SOC) in TMDC gives rise to the spin splitting in conduction band, leading to the opposite effective magnetic field in K and K' valley. The intervalley relaxation process exists for the in-plane electron spin, which is described as the DP mechanism [15]. The electron-electron interactions reduce the spin relaxation time with the increase of electron density. This spin relaxation time is related to the Fermi energy E_{F}, which is determined by the background charge density, can be written as:

$$\tau_{\mathrm{s}}\left(k_{\mathrm{F}}\right) \propto \ln\left(E_{\mathrm{F}} / k_{B}T\right) / E_{\mathrm{F}} \tag{S17}$$

where the k_{F} and k_{B} represent the Fermi wave vector and the Boltzmann constant, respectively. The trend of the calculated electron spin relaxation time versus gate voltages is shown in Supplementary Figure 15 (a). The background charge density is estimated to be at 10^{11} to 10^{12} cm^{-2} from NCR to highly charged region. As can be seen, the simulated electron spin relaxation is suppressed in NCR, and decreases evidently with increasing charge density.

ii) BAP mechanism

As for the spin relaxation of holes, the electron-hole exchange interaction is an efficient route, described as the BAP mechanism. The BAP mechanism has only been considered in heavily p-doped conventional semiconductors [14]. However, due to the increased Coulomb interactions, this exchange mechanism has been used in reduced 2D TMDC. And the exchange time is inversely proportional to background charge density, determined by the following equation[16]:

$$\frac{1}{\tau_{\mathrm{s,ex}}} \sim \Delta_{\mathrm{ex}} \frac{\Delta_{\mathrm{ex}}}{E_{\mathrm{B}}} |\psi(0)|^4 \, n a_B^2 \frac{a_{\mathrm{B}}\sqrt{2m_{\mathrm{h}}k_B T}}{\hbar} \tag{S18}$$

where n stands for the charge density. The value of parameters is listed in Supplementary Table 3. As can be seen in Supplementary Figure 15 (b), both the electron and hole spin relaxation processes are enhanced with the increase of background charge density, leading to the longest persistence of trion polarization in NCR.

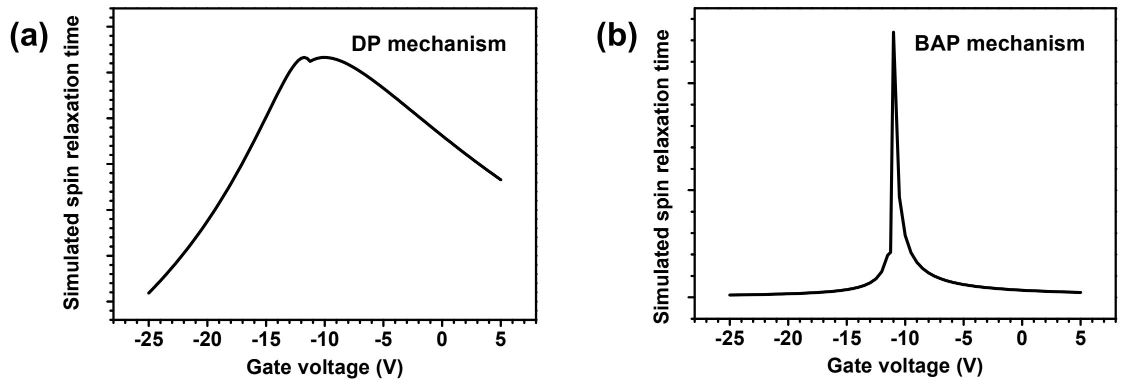

Supplementary Figure 15

Calculated spin relaxation time as a function of gate voltages. The spin relaxation time is estimated considering of DP relaxation (**a**) and BAP relaxation (**b**) mechanism. The spin relaxation time is plotted to show the trend with gate control, with no respect to exact values.

Supplementary Table 3

Definition and value of the parameters in calculation of spin relaxation time

Parameters	Definition	Value
Δ_{ex}	exchange splitting	1 meV [16]
E_{B}	exciton binding energy	500 meV [4]
E_{F}	Fermi energy	0.1 – 5 meV
$\psi(0)$	e-h overlap amplitude	1 [16]
α_B	Bohr radius	1 nm [16]

Supplementary Note 17. Comparison with other reports

We compare the CW-PL DVP and intervalley scattering time results with other TMDC MLs. Supplementary Table 4 shows the detailed test conditions and results in other works[2,6,16–34].

Supplementary Table 4

Comparison of the DVP (P) and valley scattering time (τ) in different TMDC MLs.

TMDC	Exciton (X) Trion (T)	Methods	T (K)	ΔE (meV)	P (%)	τ (ps)	Ref
WS_2	X	CWPL	10 - 300	50	40 - 10 %	-	30
	T	CWPL	300	~ 100	20 - 40 %	-	22
	X	TRPL	10	-	16.2 %	2.5	19
WSe_2	X	CWPL	4.2	97	69 %	-	32
	T				51 %		
	X	CWPL	30	50 - 590	50 - 35.7 %	-	33
	T				64 - 46 % (T^-) 36 - 23 % (T^+)		
	X	TRKR	4 - 125	0 - 200	33 %	6 - 1.5	21
	T				23 %	-	
	T	Pump-probe	13	~ 0	-	~ ps (intervalley T) >> 25 ps (intravalley T)	31
	X	TRKR	70	90	24 %	1.8 ± 0.3	20
	T					80 ± 14	
	Free carriers					2.4 ± 1 ns	
	T	TRPL	4	140	-	1 ns	18
MoS_2	X	CWPL	14	~ 50	100 %	-	16
	X	CWPL	20	0 - 250	100 - 0 %	-	6
	X	CWPL	10 - 90	~ 100	32 ± 2 %	-	35
	T	CWPL	15	~ 0	75 %	1 ns*	23
	X	TRPL	4 - 300	~ 100	~ 50 - 40 %	-	28

	X	TRFR TRCD	77	~ 5 - 50	-	4.8 ± 0.2	34
$MoSe_2$	X	CWPL	20	3	84 %	-	6
	T				79 %	-	
	X	CWPL	4.2	230	9 % (6.7 T)	-	24
	T				14 % (6.7 T)	-	
	X	CWPL	5	70 - 200	22 ± 5 %	-	2
$MoSe_2$	X	Pump-probe	300	120	-	0.36 ± 0.05 (fast) 9 ± 3 (slow)	17
	X	TRPL	4	100 - 206	~ 4 %	-	29
	X	Pump-probe	5	~0			27
$MoTe_2$	X	CWPL	4.2	~ 400	78 % (29 T)	-	25
	T				36 % (29 T)		
	T	CWPL	10	~ 500	~ 30 % (10 T)		26
	X	Pump-probe	4	< 24	38 %	~ 2.17 ± 0.2	**This work**
	T				33 %	1.39 ± 0.26 ns (maintenance> 600 ps)	

Notes:

*: the results are calculated from PL polarization and PL lifetime instead of direct observation.

TRKR, TRFR and TRCD denotes for time-resolved Kerr rotation, Faraday rotation, Circular dichroism, respectively.

Supplementary References:

1. Maialle, M. Z., De Andrada E Silva, E. A. & Sham, L. J. Exciton spin dynamics in quantum wells. *Phys. Rev. B* **47**, 15776–15788 (1993).
2. Kioseoglou, G., Hanbicki, A. T., Currie, M., Friedman, A. L. & Jonker, B. T. Optical polarization and intervalley scattering in single layers of MoS_2 and $MoSe_2$. *Sci. Rep.* **6**, 25041 (2016).

3. Baranowski, M. *et al.* Dark excitons and the elusive valley polarization in transition metal dichalcogenides. *2D Mater.* **4**, 025016 (2017).
4. Robert, C. *et al.* Excitonic properties of semiconducting monolayer and bilayer $MoTe_2$. *Phys. Rev. B* **94**, 155425 (2016).
5. Ramasubramaniam, A. Large excitonic effects in monolayers of molybdenum and tungsten dichalcogenides. *Phys. Rev. B* **86**, 115409 (2012).
6. Tornatzky, H., Kaulitz, A. M. & Maultzsch, J. Resonance Profiles of Valley Polarization in Single-Layer MoS_2 and $MoSe_2$. *Phys. Rev. Lett.* **121**, 167401 (2018).
7. Saha, M. N. On a physical theory of stellar spectra. *Proc. R. Soc. London. Ser. A, Contain. Pap. a Math. Phys. Character* **99**, 135–153 (1921).
8. Robert, C. *et al.* Spin/valley pumping of resident electrons in WSe_2 and WS_2 monolayers. *Nat. Commun.* **12**, 5455 (2021).
9. Yang, J. *et al.* Robust Excitons and Trions in Monolayer $MoTe_2$. *ACS Nano* **9**, 6603–6609 (2015).
10. Portella-Oberli, M. T. *et al.* Dynamics of trion formation in InxGa1-xAs quantum wells. *Phys. Rev. Lett.* **102**, 096402 (2009).
11. Lampert, M. A. Mobile and Immobile Effective-Mass-Particle Complexes in Nonmetallic Solids. *Phys. Rev. Lett.* **1**, 450–453 (1958).
12. Wang, Z. *et al.* Excitonic complexes and optical gain in two-dimensional molybdenum ditelluride well below the Mott transition. *Light Sci. Appl.* **9**, 39 (2020).
13. Singh, A. *et al.* Trion formation dynamics in monolayer transition metal dichalcogenides. *Phys. Rev. B* **93**, 041401 (2016).
14. Žutić, I., Fabian, J. & Das Sarma, S. Spintronics: Fundamentals and applications. *Rev. Mod. Phys.* **76**, 323–410 (2004).
15. Wang, L. & Wu, M. W. Electron spin relaxation due to D'yakonov-Perel' and Elliot-Yafet mechanisms in monolayer MoS_2: Role of intravalley and intervalley processes. *Phys. Rev. B* **89**, 115302 (2014).
16. Mak, K. F., He, K., Shan, J. & Heinz, T. F. Control of valley polarization in monolayer MoS_2 by optical helicity. *Nat. Nanotechnol.* **7**, 494–498 (2012).
17. Kumar, N., He, J., He, D., Wang, Y. & Zhao, H. Valley and spin dynamics in $MoSe_2$ two-dimensional crystals. *Nanoscale* **6**, 12690–12695 (2014).

18. Wang, G. *et al.* Valley dynamics probed through charged and neutral exciton emission in monolayer WSe_2. *Phys. Rev. B* **90**, 075413 (2014).
19. Ye, Y. *et al.* Electrical generation and control of the valley carriers in a monolayer transition metal dichalcogenide. *Nat. Nanotechnol.* **11**, 598–602 (2016).
20. Yan, T., Yang, S., Li, D. & Cui, X. Long valley relaxation time of free carriers in monolayer WSe_2. *Phys. Rev. B* **95**, 241406 (2017).
21. Zhu, C. R. *et al.* Exciton valley dynamics probed by Kerr rotation in WSe_2 monolayers. *Phys. Rev. B* **90**, 161302 (2014).
22. Hanbicki, A. T. *et al.* High room temperature optical polarization due to spin-valley coupling in monolayer WS_2. *AIP Adv.* **6**, 55804 (2016).
23. Zhang, W. *et al.* Bright and highly valley polarized trions in chemically doped monolayer MoS_2. *Appl. Phys. Express* **13**, 35002 (2020).
24. Macneill, D. *et al.* Breaking of valley degeneracy by magnetic field in monolayer $MoSe_2$. *Phys. Rev. Lett.* **114**, 037401 (2015).
25. Arora, A. *et al.* Valley Zeeman splitting and valley polarization of neutral and charged excitons in monolayer $MoTe_2$ at high magnetic fields. *Nano Lett.* **16**, 3624–3629 (2016).
26. Smoleński, T. *et al.* Valley pseudospin relaxation of charged excitons in monolayer $MoTe_2$. *J. Phys. Condens. Matter* **33**, 025701 (2020).
27. Rodek, A. *et al.* Local field effects in ultrafast light-matter interaction measured by pump-probe spectroscopy of monolayer $MoSe_2$. *Nanophotonics* **10**, 2717–2728 (2021).
28. Lagarde, D. *et al.* Carrier and polarization dynamics in monolayer MoS_2. *Phys. Rev. Lett.* **112**, 047401 (2014).
29. Wang, G. *et al.* Polarization and time-resolved photoluminescence spectroscopy of excitons in $MoSe_2$ monolayers. *Appl. Phys. Lett.* **106**, 112101 (2015).
30. Zhu, B., Zeng, H., Dai, J., Gong, Z. & Cui, X. Anomalously robust valley polarization and valley coherence in bilayer WS_2. *Proc. Natl. Acad. Sci. U. S. A.* **111**, 11606–11 (2014).
31. Singh, A. *et al.* Long-Lived Valley Polarization of Intravalley Trions in Monolayer WSe_2. *Phys. Rev. Lett.* **117**, 257402 (2016).
32. Chakraborty, C., Mukherjee, A., Qiu, L. & Vamivakas, A. N. Electrically tunable valley polarization and valley coherence in monolayer WSe_2 embedded in a van der Waals heterostructure. *Opt. Mater. Express* **9**, 1479 (2019).

33. Jones, A. M. *et al.* Optical generation of excitonic valley coherence in monolayer WSe_2. *Nat. Nanotechnol.* **8**, 634–638 (2013).
34. Dal Conte, S. *et al.* Ultrafast valley relaxation dynamics in monolayer MoS_2 probed by nonequilibrium optical techniques. *Phys. Rev. B* **92**, 235425 (2015).
35. Zeng, H., Dai, J., Yao, W., Xiao, D. & Cui, X. Valley polarization in MoS_2 monolayers by optical pumping. *Nat. Nanotechnol.* **7**, 490–493 (2012).
